# Multi-omics colocalization with genome-wide association studies reveals a context-specific genetic mechanism at a childhood onset asthma risk locus

**DOI:** 10.1186/s13073-021-00967-y

**Published:** 2021-10-10

**Authors:** Marcus M. Soliai, Atsushi Kato, Britney A. Helling, Catherine T. Stanhope, James E. Norton, Katherine A. Naughton, Aiko I. Klinger, Emma E. Thompson, Selene M. Clay, Soyeon Kim, Juan C. Celedón, James E. Gern, Daniel J. Jackson, Matthew C. Altman, Robert C. Kern, Bruce K. Tan, Robert P. Schleimer, Dan L. Nicolae, Jayant M. Pinto, Carole Ober

**Affiliations:** 1 Departments of Human Genetics, University of Chicago, Chicago, IL USA; 2 Committee on Genetics, Genomics and Systems Biology, University of Chicago, Chicago, IL USA; 3 Departments of Medicine, Northwestern University Feinberg School of Medicine, Chicago, IL USA; 4 Division of Pediatric Pulmonary Medicine, UPMC Children’s Hospital of Pittsburgh, University of Pittsburgh, Pittsburgh, PA USA; 5 Department of Pediatrics, University of Wisconsin, School of Medicine and Public Health, Madison, WI 53706 USA; 6 Division of Allergy and Infectious Diseases, Department of Medicine, University of Washington, Seattle, WA USA; 7 Systems Immunology Program, Benaroya Research Institute, Seattle, WA USA; 8 Department of Otolaryngology-Head and Neck Surgery, Northwestern University Feinberg School of Medicine, Chicago, IL USA; 9 Department of Statistics, University of Chicago, Chicago, IL USA; 10 Department of Surgery, University of Chicago, Chicago, IL USA

## Abstract

**Background:**

Genome-wide association studies (GWASs) have identified thousands of variants associated with asthma and other complex diseases. However, the functional effects of most of these variants are unknown. Moreover, GWASs do not provide context-specific information on cell types or environmental factors that affect specific disease risks and outcomes. To address these limitations, we used an upper airway epithelial cell (AEC) culture model to assess transcriptional and epigenetic responses to rhinovirus (RV), an asthma-promoting pathogen, and provide context-specific functional annotations to variants discovered in GWASs of asthma.

**Methods:**

Genome-wide genetic, gene expression, and DNA methylation data in vehicle- and RV-treated upper AECs were collected from 104 individuals who had a diagnosis of airway disease (*n*=66) or were healthy participants (*n*=38). We mapped *cis* expression and methylation quantitative trait loci (*cis*-eQTLs and *cis*-meQTLs, respectively) in each treatment condition (RV and vehicle) in AECs from these individuals. A Bayesian test for colocalization between AEC molecular QTLs and adult onset asthma and childhood onset asthma GWAS SNPs, and a multi-ethnic GWAS of asthma, was used to assign the function to variants associated with asthma. We used Mendelian randomization to demonstrate DNA methylation effects on gene expression at asthma colocalized loci.

**Results:**

Asthma and allergic disease-associated GWAS SNPs were specifically enriched among molecular QTLs in AECs, but not in GWASs from non-immune diseases, and in AEC eQTLs, but not among eQTLs from other tissues. Colocalization analyses of AEC QTLs with asthma GWAS variants revealed potential molecular mechanisms of asthma, including QTLs at the *TSLP* locus that were common to both the RV and vehicle treatments and to both childhood onset and adult onset asthma, as well as QTLs at the 17q12-21 asthma locus that were specific to RV exposure and childhood onset asthma, consistent with clinical and epidemiological studies of these loci.

**Conclusions:**

This study provides evidence of functional effects for asthma risk variants in AECs and insight into RV-mediated transcriptional and epigenetic response mechanisms that modulate genetic effects in the airway and risk for asthma.

**Supplementary Information:**

The online version contains supplementary material available at 10.1186/s13073-021-00967-y.

## Background

The respiratory tract is a complex organ system that is constantly exposed to the external environment including inhaled microbes, many of which have infectious potential and cause disease. Respiratory viruses in particular are associated with both disease onset and adverse outcomes in individuals with established respiratory illnesses. Specifically, RV, the cause of the common cold, account for over 50% of respiratory tract infections [1, 2] and are a major contributor to the onset of asthma in childhood and of asthma exacerbations in children and adults [3, 4]. Despite the important role RV plays in asthma pathophysiology, little is known about how host genetics may mediate the effects of RV on asthma onset.

Since the first asthma GWAS in 2007 [5], over 150 asthma susceptibility loci have been reported at genome-wide levels of significance (*p*< 5 × 10^−8^) (e.g., [6–9]), with a region at 17q12-21 remaining the most replicated and most significant childhood onset asthma locus (reviewed in [10]). Subsequent studies showed that variants at this locus were most significantly associated with asthma in children with early life respiratory tract infections [11, 12] or exclusively in children with RV wheezing illnesses in early life [13]. However, interpretation of these findings has been incomplete due to the absence of biological context in which to interpret them. For example, GWASs do not generally consider tissue- or environment-specific effects, or gene by environment interactions. Moreover, most genome-wide epigenetic studies of exposures (e.g., [14–18]) or of asthma-related traits (e.g., [19–24]) have not integrated their findings with GWAS. Only a few studies have formally integrated asthma GWAS and epigenetic studies in airway tissues (e.g., [25–27]), but none have considered genotype effects on genome-wide epigenetic and transcriptional responses to RV infection with asthma GWASs.

A challenge in interpreting GWAS results and prioritizing candidates for further studies is that over 90% of disease-associated variants are located in non-protein-coding regions of the genome [28] and are enriched for chromatin signatures of enhancers [28, 29] and for expression quantitative trait loci (eQTLs) [30–32]. These combined observations suggest that some of these SNPs are likely to be causal variants, underlying disease pathophysiology through their effects on gene regulation. However, identifying specific causal variants and their target genes at associated loci has been challenging, and the functions of most SNPs associated with diseases in GWASs remain unknown. While databases such as GTEx, ENCODE, and ROADMAP have been used to annotate GWAS SNPs and predict molecular mechanisms through which risk variants affect disease phenotypes [32–35], they do not include all cell types relevant to all diseases or information on environmental exposures that influence disease outcomes. As a result, annotations of asthma GWAS variants have been largely limited to studies in transformed B cell lines, blood (immune) cells, whole lung tissue, or skin (e.g., refs. [5–8, 36, 37]).

In vitro cell models provide an opportunity to address these limitations by characterizing genetic and molecular responses to environmental exposures in cells from disease-relevant tissues and identifying genotypes that modify these responses [38, 39]. In vitro functional studies of airway epithelium have been used to characterize gene pathways affected by environmental disease modifiers of asthma (e.g., refs. [14, 40–42]), and to identify functional variants that contribute to asthma pathogenesis in *ex vivo* airway epithelial cells (AECs), including eQTLs [43–46] and methylation QTLs (meQTLs) [14, 19, 25]). However, no studies to date have formally integrated molecular QTLs in AECs exposed to different conditions with asthma GWAS results. Joint analysis of datasets (e.g. eQTLs, meQTLs, and GWASs) can identify variants associated with both disease risk and molecular traits as candidate causal variants that contribute to mechanisms of disease pathophysiology. To this end, a multi-trait colocalization method (moloc) [47] was recently developed to integrate summary data from GWAS and multiple molecular QTL datasets and identify candidate regulatory drivers of complex phenotypes.

Here, we report the results of a multi-omics colocalization study to identify condition-specific regulatory effects of asthma risk variants. Because the upper airway epithelium is the primary site of RV infection, we considered transcriptional and epigenetic responses to RV in AECs as a contextual model of asthma pathophysiology. We first demonstrated a specific enrichment of childhood onset asthma GWAS SNPs among airway epithelial QTLs, consistent with the important role that the epithelial barrier plays in the inception of asthma in childhood [6, 48, 49]. Our integrative multi-omics approach then revealed a molecular mechanism shared between childhood onset and adult onset asthma in the *TSLP* gene at chromosome 5q22, and an RV-specific mechanism for childhood onset asthma at the 17q12-21 asthma locus. Overall, our results revealed molecular mechanisms of asthma pathogenies in airway epithelium, some of which were agnostic to age of asthma onset and some of which were specific to childhood onset asthma.

## Methods

### Sample collection and composition

Study participants were recruited between March 2012 and August 2015 [50], and nasal specimens were collected as part of routine endoscopic sinonasal surgeries at Northwestern University Feinberg School of Medicine. AECs were obtained as part of the Chronic Rhinosinusitis Integrative Studies Program (CRISP) [50]. Because of the relevance of the airway epithelium in RV infection and asthma, we leveraged the genomics information collected from these cells to gain a functional understanding of the genetics of molecular responses to RV infection. AECs were obtained by brushing the uncinate process collected from 104 individuals at elective surgery for chronic rhinosinusitis (CRS) or other unrelated indications (adenoidectomy, dentigerous cysts, septoplasty, and tonsillectomies) at Northwestern University. These samples were collected from 63 males, 41 females, ages 18–73 years old (mean age 44), and self-reported ethnicities as White (64%), Black (17%), Hispanic (13%), and more than one ethnicity (6%). Of the 104 subjects, 49 had a current diagnosis of CRS; 27 of the 49 CRS subjects and 16 of the 55 non-CRS subjects had a current or previous physician reported diagnosis of asthma). Study participants were determined to have CRS if they met the European Position Paper on the Primary Care Diagnosis and Management of Rhinosinusitis and Nasal Polyps (EPOS) criteria [51]. DNA from whole blood was used for genotyping; if a blood sample was not available, genotyping was performed in DNA extracted from their AECs. A summary of the study design and sample composition is shown in Additional file 1: Fig. S1.

### Upper airway epithelial cell culture and RV treatment

AECs were collected from the uncinate tissue with a Rhino-Pro, nasal mucosal curette (Arlington Scientific, Inc.). After isolation, cells were washed with Dulbecco’s phosphate-buffered saline (dPBS) and immediately cultured in a Falcon 6-well plate (product number 353046) in bronchial epithelial cell growth medium (Lonza, BEGM BulletKit, catalog number CC-3170) to near confluence, and then frozen at − 80 °C for no more than three days and finally stored in liquid nitrogen between 8 and 1075 days. Cells were subsequently thawed and cultured in collagen-coated (PureCol, INAMED BioMaterials, catalog number 5409, 3 mg/mL, 1:15 dilution) tissue culture plates (6 wells of 2x Falcon 12-well clear flat bottom plates [product number 353043]) using BEGM overnight at 37 °C and 5% CO_2_. In preparation for RV (HRV-16) infection/stimulation, plates at 50–60% confluency were incubated overnight in BEGM without hydrocortisone (HC) followed by a 2-h RV infection at a multiplicity of infection (MOI) of 2 and vehicle treatment (bronchial epithelial cell basal medium (BEBM) + gentamicin/amphotericin) at 33 °C (low speed rocking, ~ 15 RPM). RV- and vehicle-treated cells were washed and then were cultured at 33 °C for 46 h (48 h total) in BEGM without HC. Cell viability was determined based on trypan blue staining or a LDH assay. Paired samples were excluded if the viability of the vehicle-treated cells exceeded 90% or there was over 30% cell death after RV treatment. Prior to our studies, we calculated the MOI as follows. A plaque-forming assay was first performed using HeLa cells to generate plaque-forming units (PFU). The average number of AECs (from 10 donors) was further used to generate the MOI by testing dose-dependent responses by RV treatment at MOI ranges between 0.2 and 10. A lower range of 2 was within the MOI range for acceptable linearity and was used for our studies.

### Genotyping and imputation

DNA was extracted from whole blood or AECs (if blood was unavailable) with the Macherey-Nagel NucleoSpin Blood L or NucleoSpin Tissue L Extraction kits, respectively, and quantified with the NanoDrop ND1000. Genotyping of all study participants was performed using the Illumina Infinium HumanCore Exome+Custom Array (550,224 SNPs). After quality control (QC) (excluding SNPs with HWE < 0.0001 by race/ethnicity, call rate < 0.95, and individuals with genotype call rates < 0.05), 529,993 markers with minor allele frequency ≥0.05 were available for analysis in 104 individuals. Ancestry principal component analysis (PCA) was performed using 676 ancestry informative markers [52] that were included on the array and overlapped with HapMap release 3 (Additional file 1: Fig. S2).

Prior to genotype imputation, individuals were categorized into two groups based on the k-means clustering of ancestry PCs, using the kmeans() function in R; individuals were grouped as European or African American based on the mean clustering of their ancestry PCs against the HapMap reference panels (Additional file 1: Fig. S2). Phasing and imputation were performed using the ShapeIt2 [53] and Impute2 [54] software packages, respectively. Variants were imputed in 5 Mb windows across the genome against the 1000 Genomes Phase 3 haplotypes (Build 37; October 2014). After imputation, genotypes from both groups were merged and QC was performed with gtool [55]. X and Y linked SNPs and autosomal SNPs that did not meet the QC criteria (info score < 0.8, MAF < 0.05, missingness > 0.05 and a probability score < 0.9) were excluded from analyses. Probability scores were converted to dosages for 6,665,552 of the remaining sites used in downstream analyses.

### RNA extraction and sequencing

Following RV and vehicle treatments, RNA and DNA were extracted from cells using the QIAGEN AllPrep DNA/RNA Kit. RNA quality and quantity were measured using the Agilent RNA 6000 Pico assay and the Agilent 2100 Bioanalyzer. RNA integrity numbers (RIN) were greater than 7.7 for all samples. cDNA libraries were constructed using the Illumina TruSeq RNA Library Prep Kit v2 and sequenced on the Illumina HiSeq 2500 System (50 bp, single-end); RNA sequencing was completed at the University of Chicago Genomics Core. Subsequently, we checked for potential sample contamination and sample swaps using the software VerifyBamID (http://genome.sph.umich.edu/wiki/VerifyBamID) [56] for cells from all 104 individuals in each treatment condition. We did not detect any cross-contamination between samples; one sample swap between two individuals was detected, which was corrected.

Sequences were mapped to the human reference genome (hg19) and reads per gene were quantified using the Spliced Transcripts Alignment to a Reference (STAR) [57] software. X,Y, and mitochondrial chromosome genes, and low count data (genes < 1CPM) were removed prior to normalization via the trimmed mean of M-values method (TMM) and variance modeling (voom) [58]. Nine samples containing < 8 M reads were removed from the analysis. Principle components analysis (PCA) identified biological and technical sources of variation in the voom-normalized RNA-seq reads for the remaining 95 samples. We identified contributors to batch and other technical effects (days in liquid nitrogen, experimental culture days, cell culture batches, RNA concentration, RNA fragment length, technician, sequencing pool). Additionally, unknown sources of variation were predicted with the Surrogate Variable Analysis (SVA) [59] package in R where 15 surrogate variables (SVs) were estimated for the samples that were included in the experiment after protecting for treatment in the full model. Voom-normalized RNA-seq data were then adjusted for technical effects (cell lysate batch, sequencing pool, technician, fragment length, RNA concentration, days frozen in liquid nitrogen, experimental culture days), smoking, SVs, sex, and ancestry PCs (1–3) using the function removeBatchEffect() from the R package limma [60]. The variance in gene expression that was associated with asthma or CRS was correlated with the SVs (Additional file 2) and was therefore removed by inclusion of SVs in our model (Additional file 3). The PCA of gene expression of the vehicle- and RV-treated samples after regression of the covariates are shown in Additional file 1: Fig. S3.

### DNA extraction and methylation profiling

Following RV and vehicle treatments, DNA was extracted from cells as described above. DNA methylation profiles for cells from each treatment were measured on the Illumina Infinium MethylationEPIC BeadChip at the University of Chicago Functional Genomics Core. Methylation data for the 104 samples were preprocessed using the minfi package [61]. Probes located on sex chromosomes and with detection *p* values greater than 0.01 in more than 10% of samples were removed from the analysis. One sample had > 5% missing probes and was therefore removed from the analysis. A preprocessing control normalization function was applied to the remaining 103 samples to correct for raw probe values or background, and a Subset-quantile Within Array Normalization (SWAN) [62] was used to correct for technical differences between the Infinium type I and type II probes. Additionally, we removed cross-reactive probes and probes within two nucleotides of a SNP with an MAF greater than 0.05 using the function rmSNPandCH() from the R package DMRcate [63].

PCA identified technical and biological sources of variation in the normalized DNA methylation datasets. Technical effects (array and cell harvest date), as well as sex, age, and smoking were significant variables in the PCA. Unknown sources of variation were predicted using the SVA package. We estimated 37 SVs after protecting for treatment. SWAN and quantile-normalized M-values were then adjusted for batch and technical effects (sample plate and cell harvest date), smoking, SVs, sex, age, and smoking using the function removeBatchEffect() in R. The variances associated with asthma or CRS in the DNA methylation data were correlated with SVs (Additional file 4) and was therefore removed by inclusion of SVs in the model (Additional file 5). The PCA of DNA methylation of the vehicle- and RV-treated samples after regression of the covariates are shown in Additional file 1: Fig. S4.

### eQTL and meQTL analyses

Prior to e/meQTL analysis, voom-transformed gene expression values and normalized methylation M-values were adjusted for technical, biological, and surrogate variables as described above. Linear regression between the imputed genotypes (MAF > 0.05) and molecular phenotypes (gene expression and methylation residuals) from each treatment condition was performed with the FastQTL [64] software package within *cis*-window sizes of ± 1 Mb (2 Mb total) of the transcription start site [32] and ± 10 kb (20 kb total) from a CpG [65] for eQTL (Additional file 6 [vehicle], Additional file 7 [RV]; FDR< 0.05) and meQTL (Additional file 8 [vehicle], Additional file 9 [RV]; FDR< 0.05) analyses, respectively. An FDR threshold of 0.05 was applied to adjust for multiple testing within each experimental dataset with the p.adjust() function in R. To confirm that neither atopy nor steroid use had significant effects on the molecular QTL results, we tested directly for interactions between atopy and genotype and between steroid use and genotype using a linear model with an interaction term using the lm() function in R for the e/meQTLs for which there was also a colocalization in any of the asthma GWASs included in this study. These tests did not identify any significant interaction effects of genotype and either atopy or steroid use or the e/meQTLs (Additional file 10: Table S1). Box plots of eQTLs and meQTLs are shown with individuals color coded to show their atopic or non-atopic status in Additional file 1: Fig. S5 and their steroid medication status in Additional file 1: Fig. S6.

### Replication studies of molecular QTLs

Replication studies of the e/meQTLs were performed with molecular QTL datasets in *ex vivo* AECs from two studies. In the first dataset, we looked for overlap between our results and cis-eQTLs and -meQTLs for genes at asthma GWAS loci in *ex vivo* nasal epithelial cells from 477 Puerto Rican children described by Kim et al [66]. In the second dataset, we compared our results to unpublished eQTLs and meQTLs in *ex vivo* nasal epithelial cells from 11-year-old children in the Urban Environment and Childhood Asthma (URECA) birth cohort study [67].

### Multivariate adaptive shrinkage analysis (mash)

An Empirical Bayes method of multivariate adaptive shrinkage was applied separately to the eQTL and meQTL data sets as implemented in the R statistical package, mashr (https://github.com/stephenslab/mashr) [68], to produce improved estimates of QTL effects and corresponding significance values in each treatment condition. To do this, mashr estimates patterns of similarity among eQTLs or meQTLs from each treatment condition (vehicle, RV). These patterns are used by mash to improve the accuracy of the molecular QTL effects by an empirical Bayes approach. Compared to direct comparisons between conditions, mash increases power, improves effect-size estimates, and provides better quantitative assessments of effect size heterogeneity of molecular QTLs, thereby allowing for greater confidence in effect sharing and estimates of condition-specificity [68]. As a confidence measurement of the direction of QTL effects, mash provides a “local false sign rate” (lfsr) that is the probability that the estimated effect has the incorrect sign [69], rather than the expected proportion of Type I errors as would be assessed using FDR thresholds. Mashr implements this in two general steps: (1) identification of pattern sharing, sparsity, and correlation among QTL effects, and (2) integration of these learned patterns to produce improved effects estimates and measures of significance for eQTLs or meQTLs in each treatment condition. To fit the mash model, we first estimated the correlation structure in the null test from a random dataset in which 2 M gene-SNP or CpG-SNP pairs were randomly chosen for eQTLs and meQTLs, respectively, from the FastQTL nominal pass. The data-driven covariances were then estimated using the most significant e/meQTLs in each gene or CpG from the FastQTL results. Posterior summaries were then computed for the ‘top’ eQTL and meQTL results (see [68]). The instructions found in the mashr eQTL analysis outline vignette were followed to run mash.

### Enrichment analysis

The R package, GWAS analysis of regulatory or functional information enrichment with LD correction (GARFIELD) [70], was used to quantify enrichment of GWAS SNPs among eQTLs and meQTLs and assess significance. GARFIELD leverages GWAS results with molecular data to identify features relevant to a phenotype of interest, while accounting for LD and matching for genotyped variants, by applying a logistic regression method to derive statistical significance for enrichment. For this study, molecular QTLs (union of e/meQTLs from each treatment condition) were tested for GWAS variant enrichment, estimated as odds ratios and enrichment *P* values derived at four GWAS *P* value thresholds: 10^−5^, 10^−6^, 10^−7^, and 10^−8^. To evaluate disease-specificity, we selected summary statistics from nine GWASs including three asthma GWASs (adult onset and childhood onset asthma [6], and a meta-analyzed multi-ethnic, all age of asthma onset from the Trans-National Asthma Genetic Consortium [TAGC] [7]), two allergic disease GWASs (hay fever/allergic rhinitis [71] and atopic dermatitis [72]), and four non-allergic GWASs (Alzheimer’s disease [73], atrial fibrillation [74], height [75], neuroticism [76]). Summary statistics from these nine GWASs were used for enrichment analyses of the 490,151 molecular QTLs (FDR< 0.05) combined from each treatment condition. These non-allergic GWASs were chosen based on similar population backgrounds (European), availability of summary statistics (as of 05/18), and both known to have genetic overlap with asthma (allergic diseases) and not known to have overlapping genetics with asthma (Alzheimer’s disease, atrial fibrillation, height, and neuroticism).

To assess tissue-specificity of our results, we examined eQTLs from the adrenal gland, frontal cortex, hypothalamus, ovary, and testis from the GTEx database version 7 (http://gtexportal.org) [32], and tested for enrichment in the adult onset and childhood onset and TAGC asthma GWAS SNPs among the epithelial eQTLs from our study combined across treatment conditions. GTEx data were matched with respect to sample size and number of eQTLs to those of the epithelium, with the exception of testis, which was included to show the consistency of the enrichment results despite it being an outlier with respect to both sample size, which was smaller, and number of eQTLs, which was larger. An OR > 1 and a FDR corrected *p* value threshold of < 0.05 was used as the significance threshold for enrichment; FDR adjusted *p* values were calculated using the p.adjust() function in R where “*n*” was determined by the number of tests in each respective enrichment analysis.

### Colocalization analysis

To estimate the posterior probability association (PPA) that a SNP contributed to the association signal in the GWAS as well as to the eQTL and/or meQTL, we applied a Bayesian statistical framework implemented in the R package moloc [47]. Summary data from adult onset and childhood onset asthma GWASs in the UK Biobank [6], and the TAGC multi-ethnic GWAS [7], along with eQTL and meQTL summary data from AECs within each treatment condition (described above), were included in the moloc analysis. Each colocalization analysis included summary data from a GWAS and epithelial cell eQTLs and meQTLs from each treatment condition. Because a genome-wide colocalization analysis was computationally challenging, genomic regions for colocalization were defined using GARFIELD. First, we analyzed the enrichment pattern of e/meSNPs from each treatment condition in each of the three asthma GWASs using the default package settings. Second, we extracted variants driving the enrichment signals at a GWAS *p* value threshold of 1 × 10^−4^. Regions were defined as 2 Mb windows centered around these variants. Only regions with at least 10 SNPs in common between all three datasets or “traits” (GWAS, eQTL, and meQTL) were assessed by moloc and 15 “configurations” of possible variant sharing were computed across these three traits (see [47] for more details). The PPA was computed by weighting the likelihood of the summary data given the prior probability that a SNP associates with each trait (asthma, gene expression, and DNA methylation) the moloc default prior probabilities were included in our analysis. Prior probabilities of 1 × 10^−4^, 1 × 10^−6^, and 1 × 10^−7^ were chosen for the association of one, two, or three traits, respectively, as recommended by the authors of moloc. False positive rates remain below 0.05 at a posterior probability thresholds as low as 0.30 [47]. We therefore considered PPAs ≥ 0.50 as evidence for colocalization in this study.

We performed six separate colocalization analyses for each treatment condition with each of the three asthma GWASs. Each analysis provided three possible configurations in which a variant is colocalized between the GWAS and QTLs: eQTL-GWAS pairs, meQTL-GWAS pairs, and eQTL-meQTL-GWAS triplets. Estimates of a posterior probability of association (PPA) is provided, reflecting the evidence for a colocalized SNP being causal for the associations in the GWAS and for the corresponding eQTL and/or meQTL.

### Promoter Capture Hi-C

We extracted data for promoter capture (pc)Hi-C in *ex vivo* bronchial epithelial cells reported by Helling et al. [42] to examine long-range interactions at the *ERBB2* (17q12-21) locus. These data were generated in cells from eight individuals (four asthmatics and four non-asthmatics) that were processed for pcHi-C within 24 h of harvest. Removal of technical artifacts followed by genome mapping of the pcHi-C reads were performed using HiCUP version 0.5.9. Promoter interactions for the eight samples (pooled) were mapped using CHiCAGO version 1.6.0. Interactions with a CHiCAGO score > 5 were considered significant.

### Mendelian randomization

To infer causal relationships between DNA methylation and gene expression on asthma risk for colocalized triplets, we performed Mendelian randomization (MR), a method in which genetic variation associated with modifiable exposures (i.e. DNA methylation) can be used as an instrumental variable to estimate the causal influence of an exposure on an outcome (i.e., DNA methylation on gene expression) [77]. We applied a two-stage least squares regression (2SLS) regression using the ivreg2 function [78] in R (https://www.r-bloggers.com/an-ivreg2-function-for-r/) to estimate the effects of DNA methylation (exposure) on gene expression (outcome) in each treatment condition, and used the QTL SNP in the colocalized triplets (eQTL-meQTL-GWAS) to assess the causal effects of DNA methylation on gene expression.

## Results

### Genome-wide *cis*-eQTLs and *cis*-meQTLs mapping in cultured airway epithelial cells

We performed eQTL and meQTL mapping using gene expression and DNA methylation data measured in the same cells. Because each gene/CpG-variant pair was tested for a linear regression slope that significantly deviated from 0, the estimated effects of the molecular QTLs reflected both the single-SNP effects of each molecular QTL as well as those of SNPs that are in linkage disequilibrium (LD) with the true QTL(s). Accordingly, these analyses do not differentiate between causal molecular QTLs from those in LD with the QTL. The numbers of SNPs associated with gene expression for at least one gene (eQTLs) and genes with at least one eQTL (eGenes), in either treatment, are summarized in Additional file 1: Fig. S6A. The number of SNPs associated with methylation levels at one or more CpG sites (meQTLs) and CpG sites with at least one meQTL (meCpGs), in either treatment, are shown in Additional file 1: Fig. S6B. Overall, we identified 60,428 eQTLs (40,354 and 37,566 from the vehicle- and RV-treated cells, respectively) associated with 1,710 genes, and 429,725 meQTLs (302,896 and 283,474 from the vehicle- and RV-treated cells, respectively) associated with DNA methylation at 38,942 CpGs.

To replicate the molecular QTLs in cultured AECs in our study, we utilized two data sets with eQTL and meQTL studies in *ex vivo* nasal epithelial cells (see Additional file 11). One data set was generated in 477 Puerto Rican children [66] and one data set was generated in > 246 children who were ~ 75% African American, ~ 17% Hispanic, and ~ 7% other ancestries [67]. The details of these studies are described in Additional file 11. Overall, we replicated over 20% of eQTLs, 50% of eGenes, 60% of meQTLs, and 80% of meCpGs in one or both studies.

### Estimating shared and condition-specific molecular QTL effects

**Fig. 1:**
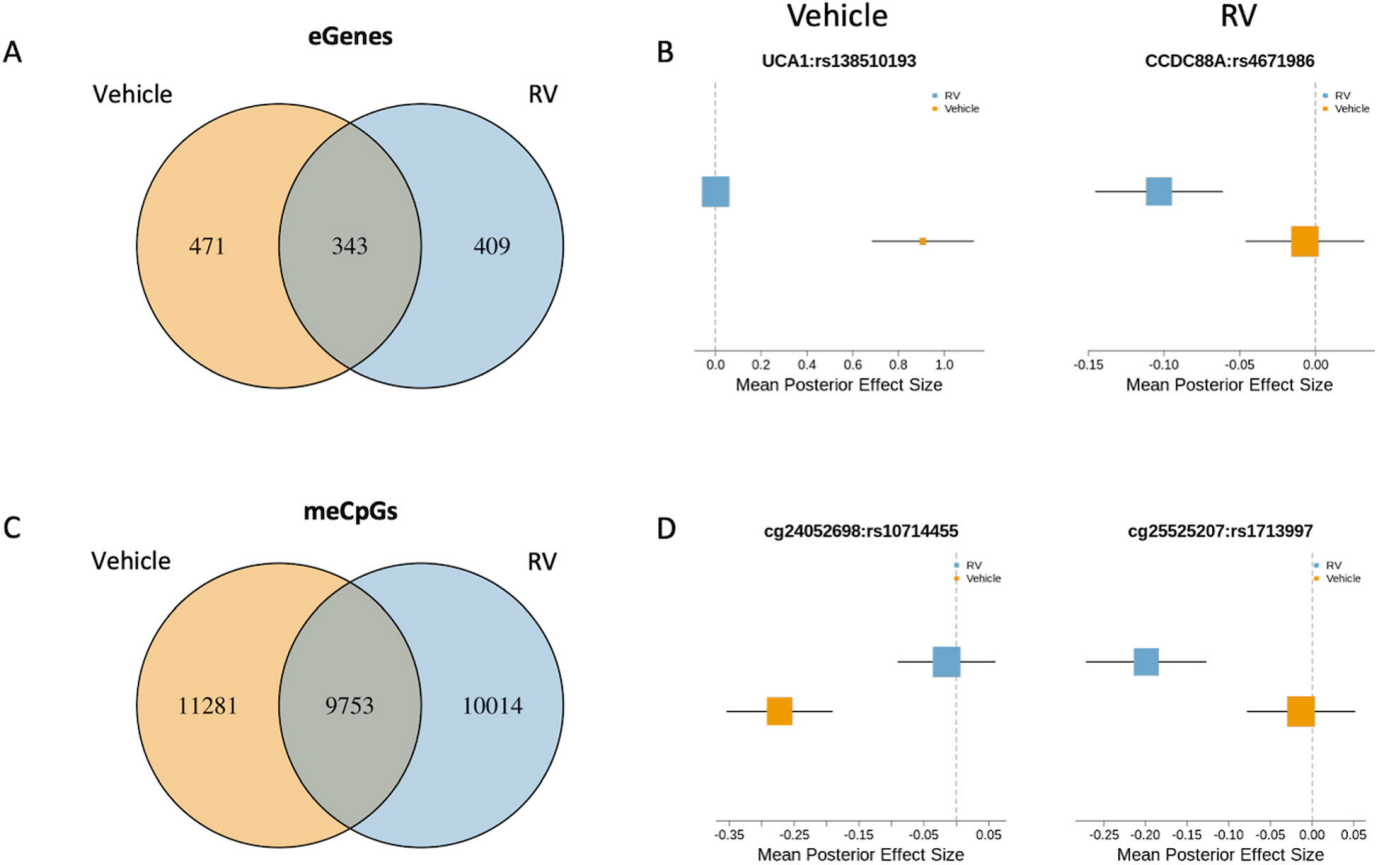
Molecular effects sharing across treatment conditions (lfsr < 0.05). Venn diagrams of eGenes (**A**) and meCpGs (**C**) shared between vehicle- and RV-treated AECs. Forest plots showing examples of vehicle- (left) and RV-specific (right) eQTLs (**B**) and meQTLs (**D**)

We first explored the impact of RV exposure on eQTLs and meQTLs by comparing RV-treated to vehicle-treated results to identify condition-specific eQTLs. For this, we analyzed the effect estimates of the most significant eQTL for each of 11,896 genes and assessed sharing of these signals among the RV and vehicle-treated cells using mash [68] (see the “Methods” section). A pairwise comparison showed that 58.3% of eQTLs were shared between RV and vehicle treatments, representing 1,223 eGenes (Fig. 1A; Additional file 12); the remaining 41.7% of eQTLs were specific to vehicle-treated (471 genes) or RV-treated (409 genes) cells. These potentially represent functional genetic variants that modify responses to viral exposure in AECs. Examples of treatment-specific eQTLs are shown in Fig. 1B.

The effect estimates of the most significant meQTL for each of 792,392 CpG sites were used to identify condition-specific DNA methylation effects, as described above for eQTLs. A pair-wise analysis of meQTLs revealed that 89.9% of meQTLs were shared between vehicle and RV treatments, representing 31,048 meCpGs (Fig. 1C; Additional file 13). This revealed a much greater proportion shared meQTLs than those observed for eQTLs. Examples of the 21,295 treatment-specific meQTLs are shown in Fig. 1D. In total, we identified 471 and 409 eGenes (41.7%) and 11,281 and 10,014 meCpGs (10.1%; both at lfsr< 0.05) that were specific to vehicle-treated or RV-treated cells, respectively.

### Molecular QTLs in the airway epithelium are enriched for asthma GWAS SNPs

To provide biological context to the identified QTLs, we used GWAS summary statistics for asthma, allergic diseases, and other presumably unrelated traits. We assessed whether the 490,151 molecular QTLs identified in our study (i.e., the union of eQTLs and meQTLs from each treatment condition at FDR< 0.05) were enriched for GWAS variants and whether these enrichments showed disease or tissue specificity. We used publicly available summary statistics from GWAS data for asthma, including childhood onset and adult onset asthma GWASs in British white individuals [6] and a multi-ethnic asthma GWAS [7], two allergic diseases (hay fever/allergic rhinitis and eczema/atopic dermatitis [72]), and four diseases without known allergic or immune etiologies (Alzheimer’s disease [73], atrial fibrillation [74], height [75], and neuroticism [76]). These analyses revealed statistically significant enrichments (OR> 1 and FDR-adjusted *P* value< 0.05; see the “Methods” section) for SNPs from all three asthma GWASs among the molecular QTLs (eQTLs and meQTLs) at each of four GWAS thresholds (Table 1), consistent with the strong epithelial cell involvement in asthma in general and with childhood onset asthma in particular. We also observed significant enrichments among the molecular QTLs for each of the two allergic disease GWASs, also consistent with the central role of epithelial cells in the regulation of allergic diseases [79] and the shared genetic architecture of asthma with these diseases [36]. In contrast, there were no significant enrichments for SNPs from the other GWASs among the epithelial cell molecular QTLs, with exception of the neuroticism GWAS SNPs, which were modestly enriched among the epithelial cell QTLs at three of the four GWAS thresholds. These results highlight the specific enrichment of asthma and allergic disease GWAS SNPs among airway epithelial molecular QTLs compared to SNPs from GWASs of diseases without known epithelial cell involvement.

**Table 1:** Enrichment estimates of airway epithelial cell eQTLs and meQTLs for GWAS SNPs. *P* values and diseases that are significant after FDR correction (see the “Methods” section) are shown in bolded type (FDR < 0.05)

	GWAS	***N***_**Cases**_	***N***_**Controls**_	***N***_**Total**_	GWAS threshold	OR	***P***	FDR
Non-allergic disease GWAS	Alzheimer’s disease [45]	47,793	328,320	376,311	1.0 × 10^−5^	0.68	2.3 × 10^−1^	2.6 × 10^−1^
					1.0 × 10^−6^	0.60	2.2 × 10^−1^	2.6 × 10^−1^
					1.0 × 10^−7^	0.64	3.5 × 10^−1^	3.7 × 10^−1^
					1.0 × 10^−8^	0.46	1.9 × 10^−1^	2.4 × 10^−1^
	Atrial fibrillation [46]	60,620	970,216	1,030,836	1.0 × 10^−5^	1.19	3.6 × 10^−1^	3.7 × 10^−1^
					1.0 × 10^−6^	1.17	5.2 × 10^−1^	5.2 × 10^−1^
					1.0 × 10^−7^	1.41	2.3 × 10^−1^	2.6 × 10^−1^
					1.0 × 10^−8^	1.70	1.3 × 10^−1^	1.7 × 10^−1^
	Height [47]	NA	NA	253,288	1.0 × 10^−5^	1.23	2.5 × 10^−1^	2.7 × 10^−1^
					1.0 × 10^−6^	1.38	1.4 × 10^−1^	1.9 × 10^−1^
					1.0 × 10^−7^	1.50	1.2 × 10^−1^	1.7 × 10^−1^
					1.0 × 10^−8^	1.51	1.6 × 10^−1^	2.0 × 10^−1^
	**Neuroticism** [48]	**130,664**	**330,470**	**461,134**	1.0 × 10^−5^	1.50	3.6 × 10^−2^	5.4 × 10^−2^
					**1.0 × 10**^−**6**^	**1.98**	**1.0 × 10**^−**2**^	**1.6 × 10**^−**2**^
					**1.0 × 10**^−**7**^	**3.53**	**2.0 × 10**^−**3**^	**3.9 × 10**^−**3**^
					**1.0 × 10**^−**8**^	**8.64**	**9.3 × 10**^−**4**^	**1.9 × 10**^−**3**^
Allergic disease GWAS	**Allergic rhinitis** [71] **(multi-ethnic)**	**26,910**	**83,424**	**110,334**	**1.0 × 10**^−**5**^	**4.82**	**1.6 × 10**^−**5**^	**9.3 × 10**^−**5**^
					**1.0 × 10**^−**6**^	**7.32**	**5.5 × 10**^−**5**^	**2.5 × 10**^−**4**^
					**1.0 × 10**^−**7**^	**5.39**	**3.2 × 10**^−**3**^	**5.5 × 10**^−**3**^
					**1.0 × 10**^−**8**^	**9.63**	**2.4 × 10**^−**3**^	**4.3 × 10**^−**3**^
	**Atopic dermatitis** [72]	**11,025**	**40,398**	**51,423**	**1.0 × 10**^−**5**^	**3.86**	**7.5 × 10**^−**4**^	**7.4 × 10**^−**4**^
					**1.0 × 10**^−**6**^	**4.54**	**5.5 × 10**^−**3**^	**2.0 × 10**^−**4**^
					**1.0 × 10**^−**7**^	**6.35**	**5.5 × 10**^−**3**^	**3.4 × 10**^−**4**^
					**1.0 × 10**^−**8**^	**7.96**	**1.7 × 10**^−**2**^	**3.1 × 10**^−**5**^
Asthma GWAS	**Asthma** [10] **(multi-ethnic; all ages of onset)**	**23,948**	**118,538**	**142,486**	**1.0 × 10**^−**5**^	**4.64**	**1.3 × 10**^−**4**^	**3.8 × 10**^−**4**^
					**1.0 × 10**^−**6**^	**6.95**	**1.7 × 10**^−**4**^	**3.8 × 10**^−**4**^
					**1.0 × 10**^−**7**^	**6.44**	**2.1 × 10**^−**3**^	**3.9 × 10**^−**3**^
					**1.0 × 10**^−**8**^	**5.16**	**8.9 × 10**^−**3**^	**1.5 × 10**^−**2**^
	**Adult onset asthma** [1]	**21,564**	**318,237**	**339,801**	**1.0 × 10**^−**5**^	**7.14**	**8.9 × 10**^−**7**^	**1.6 × 10**^−**5**^
					**1.0 × 10**^−**6**^	**8.06**	**1.4 × 10**^−**4**^	**3.9 × 10**^−**4**^
					**1.0 × 10**^−**7**^	**11.7**	**3.9 × 10**^−**4**^	**8.7 × 10**^−**4**^
					**1.0 × 10**^−**8**^	**31.8**	**1.8 × 10**^−**4**^	**4.7 × 10**^−**4**^
	**Childhood onset asthma** [1]	**9,433**	**318,237**	**327,670**	**1.0 × 10**^−**5**^	**2.83**	**3.5 × 10**^−**6**^	**3.1 × 10**^−**5**^
					**1.0 × 10**^−**6**^	**4.63**	**1.1 × 10**^−**7**^	**4.1 × 10**^−**6**^
					**1.0 × 10**^−**7**^	**4.77**	**4.4 × 10**^−**6**^	**3.1 × 10**^−**5**^
					**1.0 × 10**^−**8**^	**4.36**	**9.2 × 10**^−**5**^	**3.4 × 10**^−**4**^

To further assess the specificity of airway epithelial molecular QTLs to asthma, we compared GWAS SNP enrichments among the eQTLs in our study to those from tissues that are not known to be involved in asthma. Comparable cross-tissue data for meQTLs were not available. We tested for enrichment of asthma GWAS SNPs among eQTLs (FDR < 0.05) in five different tissues from the GTEx database (adrenal, frontal cortex, hypothalamus, ovary, testis) [32], and among the epithelial cell eQTLs from our study. We observed a significant enrichment (OR > 1 and FDR-adjusted *P*≤0.05) of childhood onset asthma (Table 2) and TAGC (Additional file 10: Table S2) GWAS SNPs among the epithelial cell eQTLs at all GWAS *P* value thresholds ≤ 1 × 10^−8^. In contrast, we did not observe enrichments of adult onset asthma GWAS SNPs among the epithelial cell eQTLs at any GWAS threshold (Additional file 10: Table S3). Except for the hypothalamus, which showed enrichment at *P* ≤ 10^−5^, no other enrichments of asthma GWAS SNPs were observed among eQTLs in other tissues, further supporting the specificity of our model and consistent with previous studies suggesting that epithelial barrier defects underlie risk for childhood onset, but not adult onset, asthma [6, 48, 49].

**Table 2:** Enrichment estimates of eQTLs for childhood onset asthma GWAS SNPs from six tissues. Significant *P* values after FDR correction are shown in bolded type (FDR ≤ 0.05)

Tissue	GWAS threshold	OR	***P***	***P***_**adj**_	***N***	***N***_**eSNP**_
Adrenal	1.0 × 10^−5^	1.31	2.2 × 10^−1^	4.7 × 10^−1^	175	588,348
	1.0 × 10^−6^	1.00	9.9 × 10^−1^	9.9 × 10^−1^		
	1.0 × 10^−7^	0.75	4.2 × 10^−1^	5.3 × 10^−1^		
	1.0 × 10^−8^	1.04	9.1 × 10^−1^	9.5 × 10^−1^		
Brain—frontal cortex	1.0 × 10^−5^	1.71	2.4 × 10^−2^	9.6 × 10^−2^	118	367,312
	1.0 × 10^−6^	1.32	3.6 × 10^−1^	5.2 × 10^−1^		
	1.0 × 10^−7^	1.23	5.7 × 10^−1^	6.8 × 10^−1^		
	1.0 × 10^−8^	1.46	3.4 × 10^−1^	5.2 × 10^−1^		
**Brain—hypothalamus**	**1.0 × 10**^−**5**^	**2.25**	**1.2 × 10**^−**3**^	**9.7 × 10**^−**3**^	**108**	**251,506**
	1.0 × 10^−6^	1.70	1.1 × 10^−1^	2.8 × 10^−1^		
	1.0 × 10^−7^	1.68	1.8 × 10^−1^	4.3 × 10^−1^		
	1.0 × 10^−8^	1.62	2.7 × 10^−1^	4.9 × 10^−1^		
Ovary	1.0 × 10^−5^	1.65	5.5 × 10^−2^	1.8 × 10^−1^	122	292,461
	1.0 × 10^−6^	1.34	3.7 × 10^−1^	5.2 × 10^−1^		
	1.0 × 10^−7^	0.94	8.9 × 10^−1^	9.5 × 10^−1^		
	1.0 × 10^−8^	0.80	6.8 × 10^−1^	7.8 × 10^−1^		
Testis	1.0 × 10^−5^	0.80	2.4 × 10^−1^	4.8 × 10^−1^	225	1,358,512
	1.0 × 10^−6^	0.69	1.0 × 10^−1^	2.8 × 10^−1^		
	1.0 × 10^−7^	0.80	4.1 × 10^−1^	5.3 × 10^−1^		
	1.0 × 10^−8^	0.75	3.3 × 10^−1^	5.2 × 10^−1^		
**Airway epithelial cells**	**1.0 × 10**^−**5**^	**3.68**	**2.8 × 10**^−**5**^	**6.8 × 10**^−**4**^	**104**	**185,407**
	**1.0 × 10**^−**6**^	**3.28**	**2.8 × 10**^−**3**^	**1.7 × 10**^−**2**^		
	**1.0 × 10**^−**7**^	**4.11**	**8.9 × 10**^−**4**^	**9.7 × 10**^−**3**^		
	**1.0 × 10**^−**8**^	**3.91**	**6.2 × 10**^−**3**^	**2.7 × 10**^−**2**^		

### Molecular QTL colocalizations with asthma risk loci

Integrating molecular QTLs with GWAS data is a powerful way to identify functional variants that may ultimately influence disease risk [80, 81] and to assign function to known disease-associated variants. We hypothesized that integrating molecular QTLs from RV- and vehicle-exposed epithelial cells with results of GWASs for asthma would reveal genetic and epigenetic mechanisms that modulate risk. Colocalization approaches directly test whether the same genetic variant (or variants in LD) underlie associations between two or more traits (e.g., gene expression and asthma), providing clues to causal disease pathways and providing biological insights to these associations.

Using this approach, we found evidence for a total of 46 unique multiple trait colocalizations (Table 3; Additional file 10: Table S4). Eleven colocalizations were detected with adult onset asthma GWAS SNPs, of which all were meQTL-GWAS pairs (11 different CpGs), and 37 colocalizations were detected with childhood onset asthma GWAS SNPs, including 22 eQTL-meQTL-GWAS triplets (13 different genes and 19 different CpGs), five eQTL-GWAS pairs (five different genes), and 10 meQTL-GWAS pairs. The latter 10 meQTL-GWAS pairs were also identified in the adult onset asthma GWAS. Ten colocalizations were detected with the TAGC GWAS SNPs, including two eQTL-meQTL-GWAS triplets, and eight meQTL-GWAS pairs associated with eight CpGs, one of which overlapped with those detected in the other two GWASs (Table 3). Overall, only a single colocalization was specific to adult onset asthma (meQTL for cg01699148); the remaining colocalizations were also observed for the childhood onset asthma GWAS SNPs. Although the triplets identified in the TAGC GWAS were not the same as those identified in the childhood onset GWAS, a gene (*ERBB2*) and CpG (cg10374813) were associated with triplets in both. Overall, these colocalizations involved 33 SNPs at 17 loci, 14 genes, and 36 CpGs. The larger number of colocalizations for childhood onset asthma GWAS SNPs is consistent both with the observation that genes at childhood onset asthma loci were most highly expressed in skin, an epithelial cell type [6], and with the enrichment of childhood onset asthma GWAS SNPs among epithelial cell eQTLs in our study. Two examples of colocalizations at prominent asthma-associated loci are described in the following sections.

**Table 3:** Number of QTL-GWAS pairs or triplets with evidence of colocalization (PPA ≥ 0.50)

GWAS	eQTL-meQTL-GWAS triplets	eQTL-GWAS	meQTL-GWAS
Adult onset asthma	0	0	11
Childhood onset asthma	22	5	10
Asthma (multi-ethnic; all ages of onset)	2	0	8
Combined (union of colocalizations)	24	5	17

### meCpGs at *TSLP* colocalize with an asthma risk variant

**Fig. 2:**
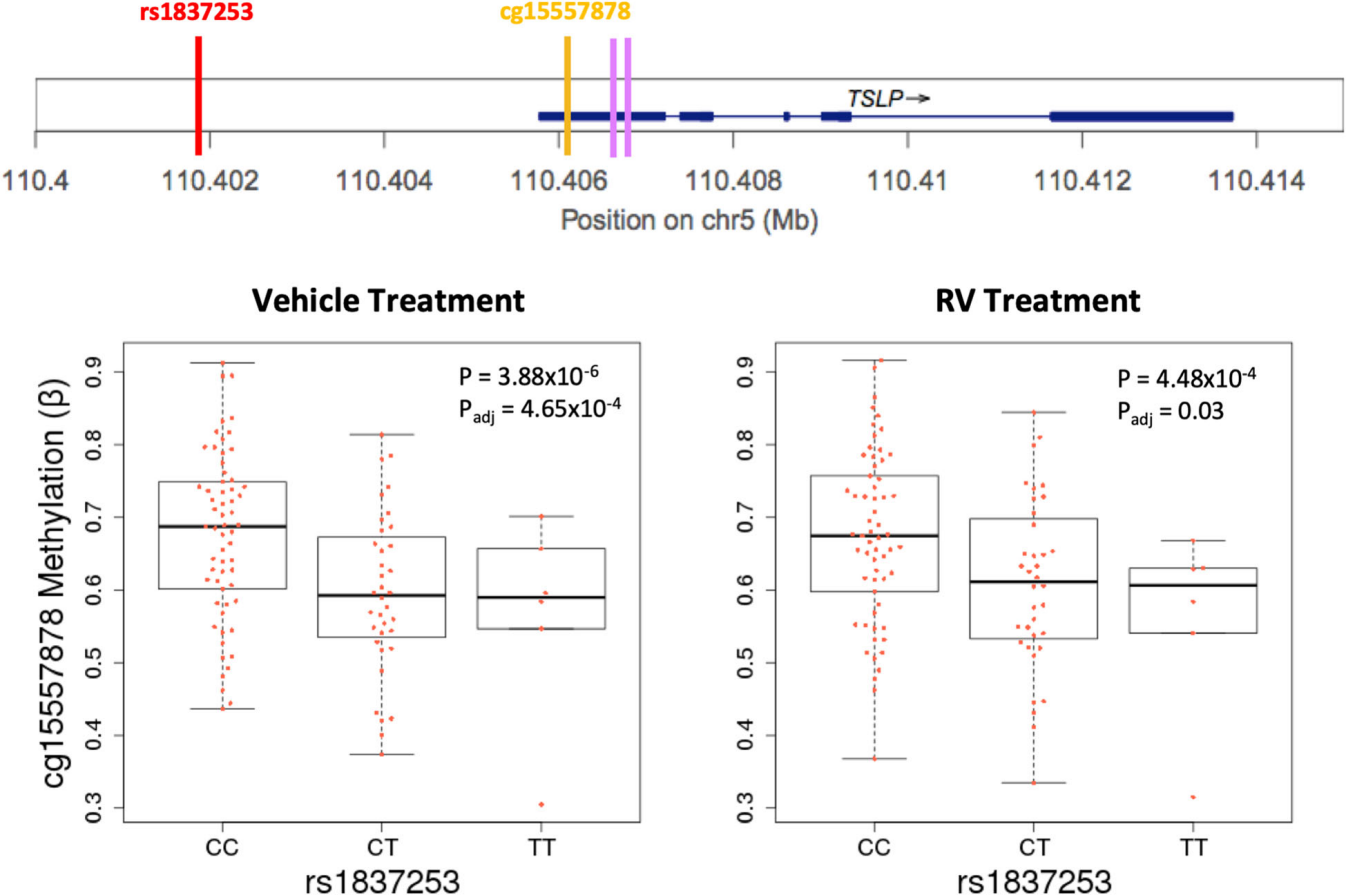
Colocalization of rs1837253 with DNA methylation levels for cg15557878 at *TSLP*. rs1837253 (red vertical bar, upper panel) is associated with DNA methylation levels at cg15557878 (orange vertical bar, upper panel). Box plots show DNA methylation levels (y-axes) for meCpGs by rs1837253 genotype (*x*-axes) in the vehicle (*β* = − 0.25; 95% CI − 0.21, − 0.30), and RV (*β* = − 0.26; 95% CI − 0.21, − 0.30) treatment conditions (lower panel). Purple vertical bars indicate the location of the remaining meCpGs co-localized with rs1837253, which was associated with asthma in all three GWASs used in the co-localization studies (*p*_GWAS_ < 10^−12^). *P* values and FDR adjusted *P* values (*P*_adj_) are shown in each box plot

To more deeply characterize the colocalizations, we first focused on the meQTL-GWAS pairs that were present in all three GWASs. Three of the 11 pairs in the adult and childhood onset GWAS and one of the eight pairs in the TAGC GWAS included an intergenic SNP (rs1837253) located 5.7 kb upstream from the transcriptional start site (TSS) of the *TSLP* gene on chromosome 5q22, encoding an epithelial cell cytokine that plays a key role in the inflammatory response in asthma and other allergic diseases [82]. rs1837253 colocalized with three meQTLs (cg10931190, cg15089387, cg15557878) in the adult onset asthma (p_GWAS_ = 2.77 × 10^−13^), childhood onset asthma (*p*_GWAS_ = 2.33 × 10^−27^), and TAGC (*p*_GWAS_ = 2.03 × 10^−25^) GWASs. An example is shown in Fig. 2; examples of all three CpGs are shown in Additional file 1 (Fig. S7). The meCpGs are located in the first (untranslated) exon (5’ UTR) of the *TSLP* gene, a region characterized as a promoter in normal human epidermal keratinocyte cells (NHEK; ROADMAP). In fact, rs1837253 was the most significant SNP at this locus in GWASs of asthma [6, 83] and of moderate-to-severe asthma [84]. In our study, the rs1837253-C asthma risk allele was associated with hypermethylation of cg15557878 in primary cultured AECs (Fig. 2; Additional file 10: Table S5) but was not associated with the expression of *TSLP* in either treatment condition (not shown). The colocalization of rs1837253 with an meQTL (cg15557878) in three separate asthma GWASs provides robust support for DNA methylation effects on asthma risk at this locus.

### Multi-trait colocalizations of molecular QTLs and asthma risk at the 17q12-21 asthma locus

To further explore the possibility that some mechanisms of asthma risk are exposure-specific, we focused on the colocalizations of eQTLs and meQTLs with asthma-associated SNPs at the 17q12-21 (17q) locus, the most significant and most highly replicated locus for childhood onset asthma (reviewed in [10]). This locus is characterized by high LD across a core region of 150 kb in non-African ancestry population, encoding at least 4 genes (including *ORMDL3* and *GSDMB*). SNPs extending both proximal (including *PGAP3* and *ERBB2*) and distal (including *GSDMA*) to the core region show less LD with those in the core region and have been implicated as potentially independent asthma risk loci. Previous studies have shown that SNPs at this extended locus are eQTLs for at least four genes (*ORMDL3*, *GSDMB*, *GSDMA*, *PGAP3*) in blood and/or lung cells [10] and that their genotype effects on risk for childhood onset asthma are modified by early life wheezing illness in general [11, 12, 85] and RV-associated wheezing illness in particular [13].

**Fig. 3:**
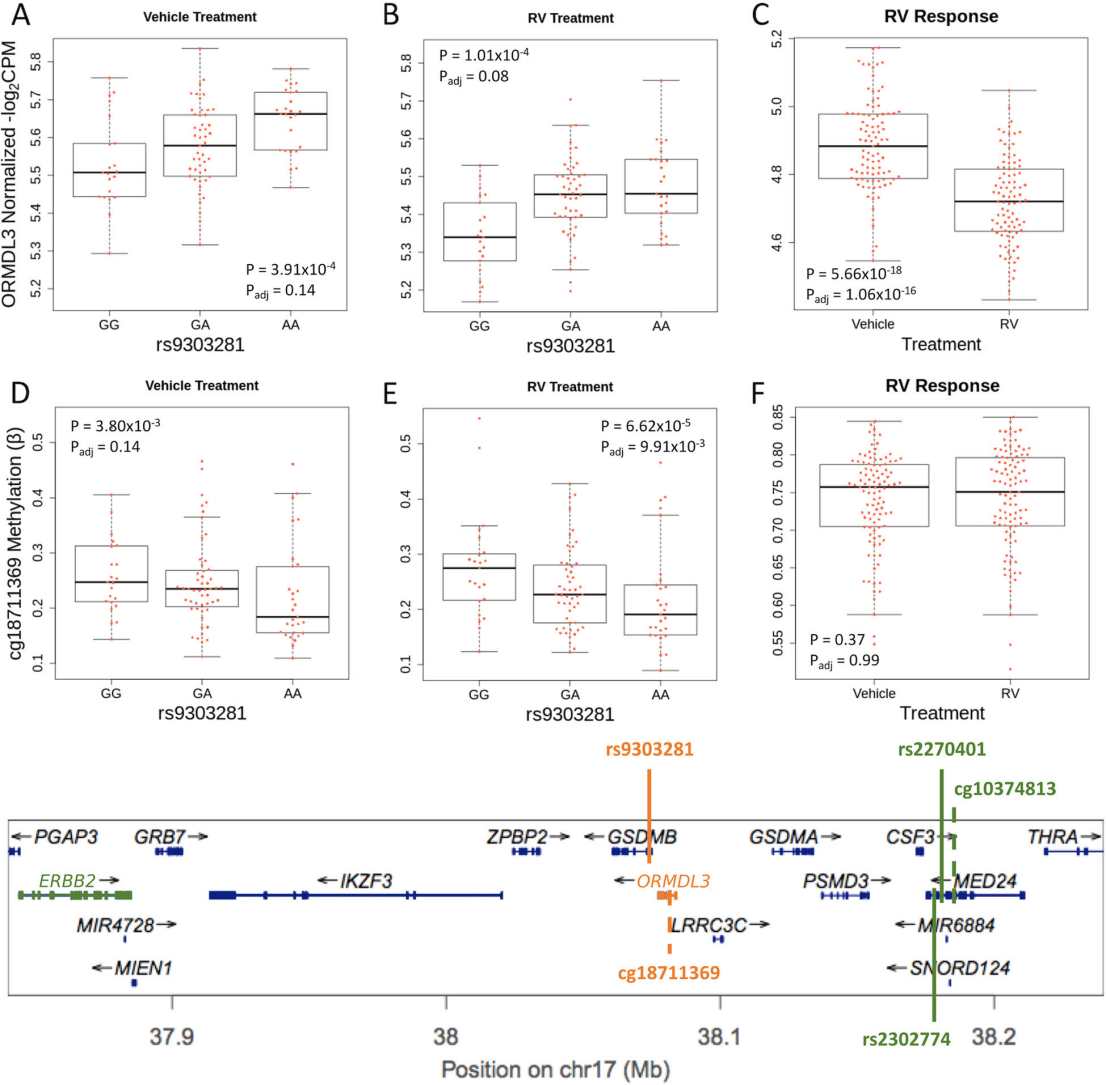
Colocalization pairs at the 17q asthma susceptibility locus. Upper panel: Box plots for the co-localized *ORMDL3* eQTL in cultured AECs treated with vehicle (**A**; *β* = 0.06; 95% CI 0.07, 0.04) and RV (**B**; *β* = 0.06; 95% CI 0.08, 0.05). *ORMDL3* gene expression decreases after treatment with RV (**C**). Box plots of the cg21230266 meQTL treated with vehicle (**D**) and RV (**E**), and methylation levels at cg21230266 (**F**). The meQTLs and overall methylation levels are similar in vehicle and RV treatments. *P* values and FDR adjusted *P* values (*P*_adj_) are shown in each box plot. Lower panel: The extended 17q12-21 locus. Co-localizations are shown by the vertical colored lines. Solid lines indicate the position of the colocalized SNP. Dashed lines indicate the location of meCpG pairs. Traits of the same co-localization are shown in the same color. The eQTL-meQTL-GWAS co-localization for *ORMDL3* is shown in orange, and the eQTL-meQTL-GWAS co-localization for *ERBB2* is shown in green

We identified four colocalizations at this locus (Fig. 3). One colocalization in the core region was identified only in the TAGC GWAS, and three at the distal end of the 17q locus was identified in both the childhood onset and TAGC GWASs. The TAGC-only colocalization was an meQTL-GWAS pair that was associated with cg10374813 and the three TAGC and childhood onset asthma colocalizations were eQTL-meQTL-GWAS triplets associated with two genes (*ORMDL3* and *ERBB2*) and two CpGs (cg18711369 and cg2270401). The TAGC triplet was colocalized only in RV-treated cells and included an eQTL for *ORMDL3* (TAGC *p*_GWAS_ = 6.20 × 10^−45^; PPA ≥ 0.50; Fig. 3A, B). The SNP (rs9303281) associated with this colocalization was located within the *GSDMB* gene body and the CpG (cg18711369) was located within the *ORMDL3* gene body (approximately 7.14 kb from rs9303281). The colocalized SNP is in LD (*r*^2^ > 0.85 in the 1000 Genomes CEU reference panel) with other previously reported asthma-associated GWAS SNPs in this region (see [10]), including some that were reported as eQTLs for *ORMDL3* and *GSDMB*, primarily in resting blood immune cells. Although the *ORMDL3* colocalization was significant only in the RV-treated cells, the eQTL for this SNP-gene pair was also detected in the vehicle treatment. These data indicate that the asthma risk allele (A) is associated with decreased DNA methylation (Fig. 3D, E) and increased expression of *ORMDL3* in both vehicle and RV-treated cells. However, whereas *ORMDL3* expression significantly decreased after exposure to RV (Fig. 3C), DNA methylation levels at cg18711369 remain unchanged with RV infection (Fig. 3F), consistent with our observation of relatively few treatment-specific meQTLs.

**Fig. 4:**
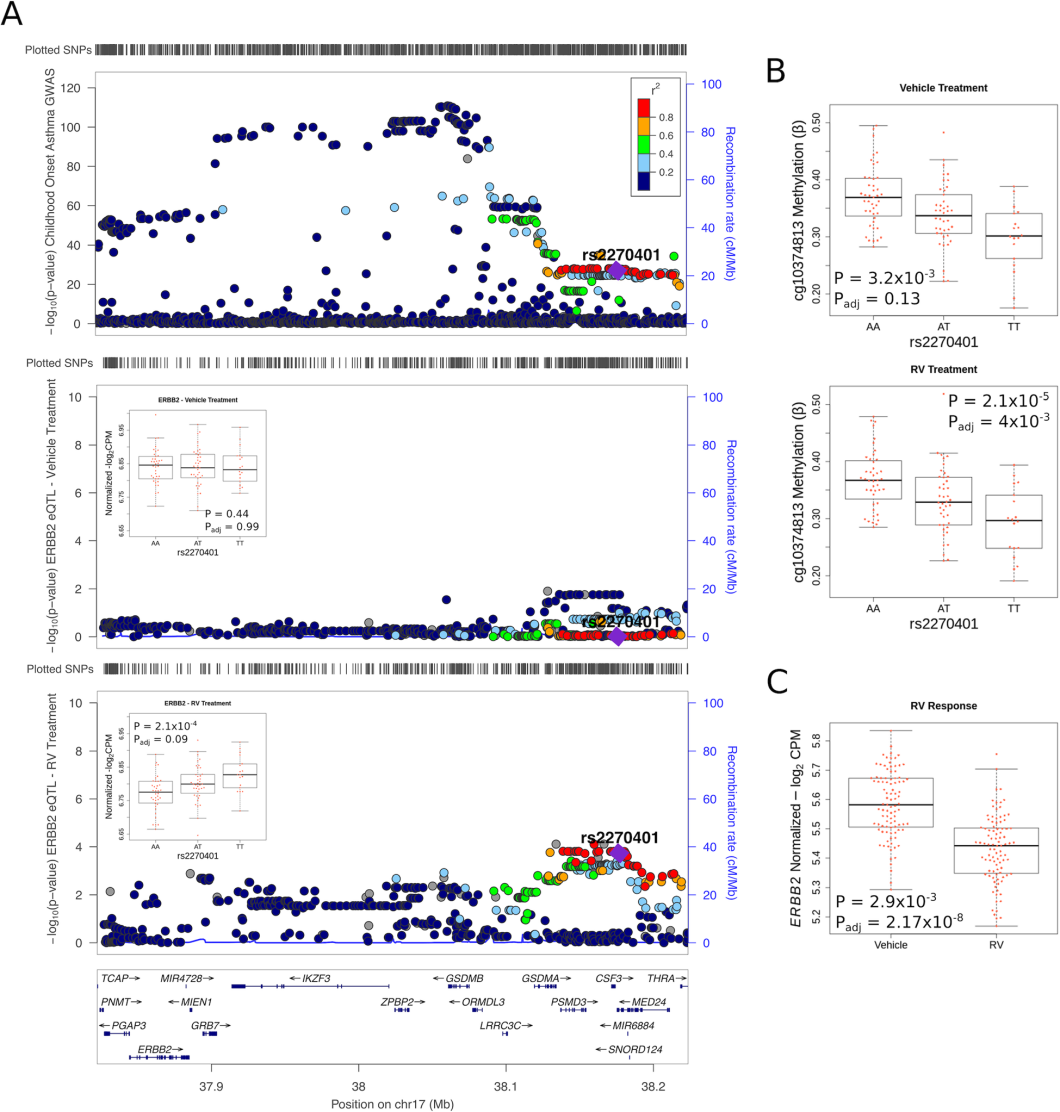
Colocalization of rs2270401 with *ERBB2* expression and DNA methylation levels for cg10374813. **A** LocusZoom plots of childhood onset GWAS results at the 17q locus showing the *ERBB2* gene at the proximal (left) end of the locus and the co-localized eQTL (rs2270401) at the distal (right) end of the locus. The SNP (rs2270401), which colocalized with associations for childhood onset asthma, *ERBB2* expression, and DNA methylation at cg10374813, is shown as a purple diamond in each of three LocusZoom plot. Upper panel: childhood onset asthma GWAS (modified from Pividori et al. 2019). Middle panel: *ERBB2* eQTLs for vehicle-treated cultured AECs. The association for *ERBB2* gene expression and rs2270401 for the vehicle treatment is shown in the box plot (*β* = − 1.16 × 10^−3^; 95% CI − 6.40 × 10^−3^, − 8.73 × 10^−3^). Lower panel: *ERBB2* eQTLs for RV-treated AECs. Boxplots for *ERBB2* gene expression by rs2270401 genotype is shown within the middle and lower LocusZoom plots. The association of *ERBB2* gene expression and rs2270401 for the RV treatment is shown in the box plot (*β* = 0.03; 95% CI 0.04, 0.02). **B** Boxplots for cg1037813 meQTLs in vehicle-treated (upper panel; *β* = − 0.07; 95% CI − 0.07, − 0.09) and RV-treated (lower panel; *β* = − 0.10; 95% CI − 0.07, − 0.12) cultured AECs. **C** *ERBB2* gene expression in vehicle-treated and RV-treated cells. *P* values and FDR adjusted *P* values (*P*_adj_) are shown in each box plot

The remaining two eQTL-meQTL-GWAS triplets spanned the entire locus (Fig. 4A, upper panel), included two eQTLs for *ERBB2*, which is located at the proximal end of the locus, more than 290 kb from the two colocalized asthma risk variants rs2270401 (childhood onset) and rs2302774 (TAGC) at the distal end of the locus. These two SNPs are in strong LD (*r*^2^=0.94 in CEU). The one colocalized meCpG (cg10374813) is in an intron of *MED24* (Fig. 4A, middle panel), located beyond the extended 17q12-21 distal locus as previously defined [10], in a region characterized by ROADMAP as an enhancer in NHEK cells. Although the meQTLs were present in both vehicle and RV treated cells (Fig. 4B upper and lower panels, respectively), the eQTLs for *ERBB2* were observed only after exposure to RV (Fig. 4A middle and lower panels show results for rs2270401). The asthma risk alleles in the childhood onset asthma (rs2270401-A) and TAGC (rs2302774-G) GWASs were associated with hypermethylation of cg10374813 in both conditions and decreased *ERBB2* expression only in RV-treated cells (Additional file 10: Table S5). Overall *ERBB2* expression decreased in response to RV exposure in AECs (Fig. 4D). That the meQTL for cg10374813 was observed in both conditions suggests an epigenetically poised chromatin state at the distal end of the locus that directly affects transcription of *ERBB2* at the proximal end of the locus after exposure to RV, and possibly to other viruses.

**Fig. 5:**
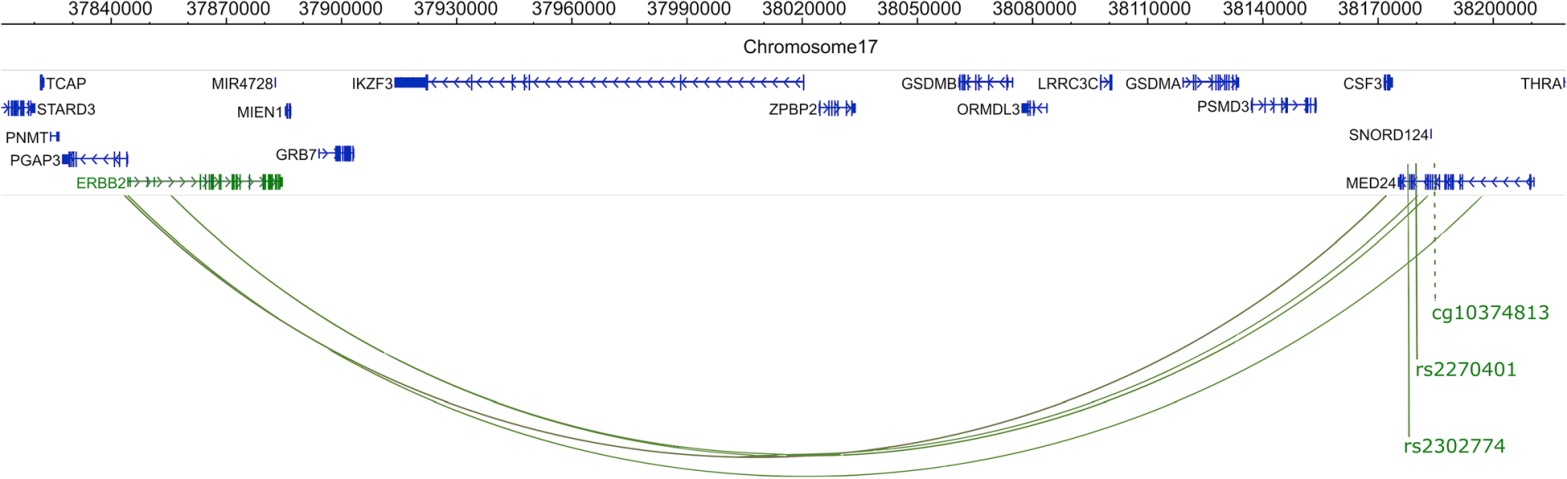
pcHi-C Interactions with *ERBB2* and *MED24* from *ex vivo* bronchial epithelial cells. pcHi-C interaction map indicating chromatin interactions (green arcs) between *ERBB2* and *MED24*. The looping is occurring between the *MED24* gene and the *ERBB2* promoters. Solid green vertical lines indicate the SNPs associated with the co-localized triplet from the childhood onset asthma (rs2270401) and TAGC (rs2302774) GWASs. The dashed vertical line shows the location of the CpG associated with this triplet. A Capture Hi-C Analysis of Genomic Organisation (CHiCAGO) score ≥ 5 was considered as evidence for chromatin interactions (range for the four loops: 5.02–7.02)

The > 290 kb distance between the promoter of *ERBB2* and its eSNPs (rs2270401, rs2302774) suggested a long-range interaction between *ERBB2* and the region harboring both cg10374813 and the asthma-associated SNPs (rs2270401, rs2302774). To examine this possibility, we used pcHi-C data from *ex vivo* primary bronchial epithelial cells [42]. These data revealed four chromatin-chromatin interactions (or loops) between the *ERBB2* promoter and the *MED24* gene (Fig. 5). These data provide empiric support for these colocalizations and suggested a potential epigenetic mechanism at the *MED24* locus that impacts the expression of *ERBB2* upon viral infection and highlights a broad regulatory landscape across the 17q12-21 asthma locus.

### Mendelian randomization of multi-trait colocalized triplets

Colocalization analyses revealed genetic variants that were associated with both asthma and molecular traits (gene expression and/or DNA methylation) but the question of causality remains unanswered. To estimate whether the effects of the asthma risk variant on gene expression are mediated by DNA methylation in colocalized triplets, we performed Mendelian randomization (MR) for each of the 24 triplets using a two-stage least squares regression model (2SLS) [78]. In each case, MR suggested a causal relationship between methylation and gene expression, indicating that the genotype effect at each of the colocalized SNPs on gene expression is mediated by DNA methylation at the colocalized meCpG (Table 4). Four of the 20 colocalizations were significant only in the RV-treated samples. Two of the RV-specific triplets involved *ERBB2* at the 17q12-21 locus, as discussed above and two were located on chromosome 11. Although the chromosome 11 colocalized SNPs (rs11227318, rs10791827) were in high LD and associated with the same CpG (cg15531562), they were associated with the expression of two different genes (*RBM14*, *PACS1*).

**Table 4:** Mendelian randomization results of gene expression and DNA methylation identified from colocalization triplets. We considered the relationship between DNA methylation and gene expression to be causal if the adjusted *P* value ≤ 0.05

SNP	SNP Position (Chr:Pos)	CpG	Gene	Vehicle		RV	
				*P*	*P*_adj_	*P*	*P*_adj_
rs34372395	1:152167407	cg15025200	*FLG-AS1*	< 1.0 × 10^−3^	< 1.2 × 10^−3^	< 1.0 × 10^−3^	< 2.8 × 10^−3^
rs34372395	1:152167407	cg23107878	*FLG-AS1*	1.0 × 10^−3^	1.2 × 10^−3^	< 1.0 × 10^−3^	< 2.8 × 10^−3^
rs1552994	1:152171461	cg09127314	*FLG-AS1*	< 1.0 × 10^−3^	< 1.2 × 10^−3^	< 1.0 × 10^−3^	< 2.8 × 10^−3^
rs1552994	1:152171461	cg21280320	*FLG-AS1*	< 1.0 × 10^−3^	< 1.2 × 10^−3^	< 1.0 × 10^−3^	< 2.8 × 10^−3^
rs1552994	1:152171461	cg02754945	*FLG-AS1*	1.0 × 10^−3^	1.8 × 10^−3^	< 1.0 × 10^−3^	< 2.8 × 10^−3^
rs1552994	1:152171461	cg26320663	*FLG-AS1*	1.0 × 10^−3^	1.8 × 10^−3^	< 1.0 × 10^−3^	< 2.8 × 10^−3^
rs1552994	1:152171461	cg13498757	*FLG-AS1*	2.0 × 10^−3^	3.1 × 10^−3^	< 1.0 × 10^−3^	< 2.8 × 10^−3^
rs7545406	1:152193286	cg26879891	*FLG*	< 1.0 × 10^−3^	< 1.2 × 10^−3^	< 1.0 × 10^−3^	< 2.8 × 10^−3^
rs2689273	3:121631451	cg19216788	*SLC15A2*	1.0 × 10^−3^	1.8 × 10^−3^	2.0 × 10^−3^	2.8 × 10^−3^
rs9822474	3:121637966	cg07193051	*SLC15A2*	< 1.0 × 10^−3^	< 1.2 × 10^−3^	2.0 × 10^−3^	2.8 × 10^−3^
rs351855	5:176520243	cg19956155	*FGFR4*	2.1 × 10^−2^	2.8 × 10^−2^	< 1.0 × 10^−3^	< 2.8 × 10^−3^
rs3807306	7:128580680	cg26616347	*IRF5*	< 1.0 × 10^−3^	< 1.2 × 10^−3^	< 1.0 × 10^−3^	< 2.8 × 10^−3^
rs11227318	11:65592935	cg15531562	*RBM4B*	1.0 × 10^−3^	1.8 × 10^−3^	5.0 × 10^−2^	5.0 × 10^−2^
*rs11227318	11:65592935	cg15531562	*RBM14*	1.2 × 10^−1^	1.3 × 10^−1^	< 1.0 × 10^−3^	< 2.8 × 10^−3^
rs10791827	11:65596546	cg15531562	*EFEMP2*	< 1.0 × 10^−3^	< 1.2 × 10^−3^	1.6 × 10^−2^	1.7 × 10^−−2^
*rs10791827	11:65596546	cg15531562	*PACS1*	8.3 × 10^-1^	8.3 × 10^−1^	1.0 × 10^−−3^	1.6 × 10^−3^
rs9303281	17:38074046	cg18711369	*ORMDL3*	1.0 × 10^−3^	1.8 × 10^−3^	1.0 × 10^−3^	1.6 × 10^−3^
*rs2270401	17:38176256	cg10374813	*ERBB2*	7.2 × 10^−1^	7.8 × 10^−−1^	1.0 × 10^−3^	1.6 × 10^−3^
*rs2302774	17:38183090	cg10374813	*ERBB2*	7.7 × 10^−1^	8.0 × 10^−1^	5.0 × 10^−3^	5.9 × 10^−3^
rs1029792	17:38808941	cg26165421	*SMARCE1*	2.1 × 10^−2^	2.8 × 10^−2^	< 1.0 × 10^−3^	< 2.8 × 10^−3^
rs7223354	17:38824672	cg02645492	*SMARCE1*	3.6 × 10^−2^	4.3 × 10^−2^	1.0 × 10^−3^	1.6 × 10^−3^
rs132920	22:41810170	cg19274703	*ACO2*	2.0 × 10^−3^	3.1 × 10^−3^	5.0 × 10^−3^	5.9 × 10^−3^
rs9607819	22:41958862	cg07830128	*PMM1*	4.0 × 10^−3^	5.9 × 10^−3^	5.0 × 10^−3^	5.9 × 10^−3^
rs5758461	22:42162189	cg10386501	*PMM1*	3.1 × 10^−2^	3.9 × 10^−2^	2.4 × 10^−2^	2.5 × 10^−2^

*Treatment-specific causal effects (FDR ≤ 0.05)

The results of the MR analyses provide orthogonal evidence for the colocalization of the triplets and novel evidence for causal inference with respect to the colocalized molecular traits (DNA methylation, gene expression). These data also reinforce arguments for epigenetic mechanisms modifying gene expression, and potentially asthma risk, in response to environmental exposures [86, 87].

## Discussion

One of the major challenges of complex disease genetics is to uncover molecular mechanisms of pathogenesis and to understand how genetic and environmental factors interact to influence risks for disease. While GWASs have identified thousands of SNPs associated with hundreds of disease phenotypes, interpretation and downstream follow-up studies have been challenging [88]. Cell models can advance our understanding of disease pathobiology through experimental testing of disease mechanisms in a controlled environment. In this multi-omics study, we leveraged an AEC model of microbial response to identify potentially functional variants with context-specific effects on transcriptional and epigenetic responses and molecular mechanisms of disease. We showed that asthma and allergy GWAS SNPs were specifically enriched among molecular QTLs in AECs compared to SNPs from other GWASs, and among AEC eQTLs compared to eQTLs from other tissues. Finally, SNPs that were molecular QTLs in our study colocalized with asthma GWAS SNPs, revealing 46 unique colocalizations that included both known asthma loci (e.g., 17q12-21 and *TSLP*) and loci that did not meet stringent criteria for genome-wide significance in the GWASs (e.g., *IRF5* and *PMM1*) (Additional file 10: Table S4).

The results of enrichment analyses further highlighted the important role of airway epithelium in asthma pathogenesis. The enrichment of childhood onset asthma and TAGC GWAS SNPs among epithelial eQTLs is particularly noteworthy, and consistent with previous studies suggesting that functional variants from disease-relevant tissues are more enriched among GWAS loci for those diseases [31, 89, 90]. The absence of enrichment of adult onset asthma GWAS SNPs among epithelial eQTLs may be due to the overall smaller effect sizes of SNPs at adult onset asthma GWAS loci compared to childhood onset asthma GWAS loci, to the less important role of epithelial cells in the pathophysiology of adult onset asthma, or to the greater heterogeneity and lesser heritability of adult onset asthma [6]. While we did not observe an enrichment of adult onset asthma GWAS SNPs among AEC eQTLs, SNPs were enriched among AEC molecular QTLs when we considered meQTLs. This suggests that DNA methylation may be a relatively more important contributor to adult onset compared to childhood onset asthma, consistent with both the greater environmental contributions to adult onset asthma [6] and the more stable nature of DNA methylation compared to gene expression across treatments in our study and possibly throughout life (e.g., [15]).

Other differences between the adult onset and childhood onset asthma GWASs were observed. For example, 11 colocalizations were detected with adult onset asthma GWAS SNPs, compared to 39 with childhood onset asthma GWAS SNPs. None of the colocalizations in the adult onset GWAS included an eQTL compared to 28 childhood onset colocalizations with eQTLs, and 10 of the 11 meQTL-GWAS pairs in the adult onset asthma GWAS were also present in the childhood onset asthma GWAS. These differences were additionally surprising because although there were 2.5 times the number of loci associated with childhood onset asthma compared to adult onset asthma in the GWASs [6], there were over 3.5 times more colocalizations in the childhood onset compared to the adult onset GWAS (39 vs. 11, respectively). These observations likely reflect the more important role of gene regulation and dysregulation in airway epithelium in the etiology of childhood onset asthma compared to adult onset asthma [48, 49]. Focusing on other asthma relevant tissues or cells (e.g., immune cells, airway smooth muscle cells) might reveal additional novel molecular mechanisms and differences between childhood onset and adult onset asthma. As with the adult onset asthma GWAS, there were fewer colocalizations using the TAGC GWAS compared to the childhood onset asthma GWAS (10 vs. 39, respectively). This may be due to the inclusion of all ages of asthma onset and multiple ethnicities, fewer reported genome-wide significant loci, and/or smaller effect sizes of the associated alleles in the TAGC GWAS. These combined factors may have reduced the power to identify colocalizations with the TAGC GWAS SNPs.

The enrichments of AEC molecular QTLs with neuroticism GWAS SNPs and of asthma GWAS SNPs with hypothalamus eQTLs were unexpected but may reflect the greater than expected cooccurrence of asthma and neuroticism [91, 92] and hypothalamic-pituitary-adrenal axis influence on the development of neuroticism [93, 94]. Whether these observations are due to the co-occurrence of these conditions or a shared genetic architecture cannot be discerned from our studies.

Our study provided mechanistic evidence for two important asthma GWAS loci: the *TSLP* and 17q12-21 loci. SNPs at the *TSLP* locus have been highly replicated in asthma GWASs, and TSLP is recognized as having an important role in asthma pathogenesis through its broad effects on innate and adaptive immune cells promoting Th2 inflammation [95]. Previous studies have shown *TSLP* to be a methylation-sensitive gene, and hypomethylation at its promoter was associated with atopic dermatitis and prenatal tobacco smoke exposure [96, 97], two asthma-associated features. Another study showed that the rs1837253-CC genotype was associated with increased excretion of TSLP in cultured AECs after exposure to polyI:C (a dsRNA surrogate of viral stimulation) [98]. Through colocalization, we further showed that the effect of rs1837253 genotype on risk for asthma may be mediated through DNA methylation levels at CpG sites in the untranslated first exon of the *TSLP* gene in AECs, suggesting an epigenetic mechanism of disease that is robust to RV and vehicle treatment and contributes to both adult and childhood onset asthma. We were unable to identify any SNPs in LD with rs1837253 (± 50 kb) with *r*^2^ > 0.12 in 1000Genomes in European or African ancestry reference panels, suggesting that this SNP may indeed be the causal asthma SNP at this important locus.

Since its discovery over a decade ago, the 17q12-21 locus has been an important focus of asthma research. Several studies have revealed the complex nature of this locus including the differences in LD structure across populations, and different gene expression patterns and eQTLs in different asthma-relevant tissues and cell types (reviewed in [10, 46]). Our AEC model of RV infection revealed additional dimensions of complexity at this locus. For example, we showed that one of the candidate genes in the core region, *ORMDL3*, is downregulated by RV in cultured AECs, and that an eQTL for *ORMDL3* colocalizes with a meQTL in a neighboring gene, *GSDMB*, and with a TAGC asthma GWAS SNP. This contrasts with a recent study [46] in *ex vivo* AECs, in which 17q12-21 core region SNPs were eQTLs for *GSDMB* but not *ORMDL3*, although the colocalized SNP in our study (rs9303281) was not included in that study. These disparate findings could be due to differences between cultured cells, which were comprised of basal epithelial cells in this study, and *ex vivo* cells, which are comprised of fully differentiated epithelial cells. Nonetheless, our study raises the possibility that downregulation of *ORMDL3* during infection with RV, and potentially other viruses, plays a role in the strong association of this locus with early life wheezing [11, 13, 99].

We also identified a long-range epigenetic mechanism at the 17q12-21 locus that has not been previously described and implicates for the first time a gene at the proximal end of this locus, *ERBB2*, in a genetic study of asthma. Colocalization and Mendelian randomization revealed a novel epigenetic mechanism through which a SNP at the distal boundary of the locus (in *MED24*) was associated with expression of *ERBB2* at the proximal boundary of the locus, only after exposure to RV. We observed this colocalized triplet in both the childhood onset and TAGC GWASs. Although the colocalizations identified different asthma associated alleles in each study, which were in strong LD (*r*^2^ = 0.94; 1000 Genomes CEU reference panel), the same epigenetic mechanism was supported by both studies. The eQTL effect on *ERBB2* expression only in RV-treated cells was mediated through a differentially methylated CpG site also in *MED24* at the distal locus, which was present in both treatment conditions and colocalized in both GWASs (Additional file 10: Table S4). The SNPs that were eQTLs for *ERBB2* in RV infected epithelial cells showed strong evidence of association with asthma (childhood onset asthma *p*_GWAS_ = 8.11 × 10^−29^ [6]; TAGC *p*_GWAS_ = 2.79 × 10^−20^ [7]), directly connecting the eQTL for *ERBB2* in RV-treated cells to asthma risk. The asthma-associated alleles, rs2270401-A and rs2302774-G, respectively, were both associated with decreased expression of *ERBB2* after RV infection (Fig. 4A), consistent with results of a study in 155 asthma cases and controls reporting an inverse correlation between *ERBB2* expression in *ex vivo* lower AECs and asthma severity [100]. These combined data suggest that decreased expression of *ERBB2*, which is associated with increased asthma severity, may be modulated by RV, the most common trigger of asthma exacerbations, via epigenetic mechanisms involving DNA methylation and long-range chromatin looping between the proximal and distal ends of this important locus. The latter was further supported by pcHi-C studies.

The significance threshold (*p* < 5 × 10^−8^) required to control the false discovery rate in GWASs likely excludes many true associations that do not reach this stringent cutoff. However, distinguishing true from false positive signals for variants among the mid-hanging fruit (e.g., *p* values between 10 and 5 and > 10^−8^) can be challenging [101]. We and others have suggested that these SNPs may be environment- or context-specific associations that are missed in GWASs that typically do not control for either [101–103]. Notably, over 52% (24 of 46) of the colocalizations in our study were with a GWAS SNP that did not meet genome-wide significance (five in the adult onset asthma, 16 in the childhood onset asthma, and three in the TAGC GWASs). This may be due to the variants having exposure-specific, tissue-specific, or endotype-specific effects, which are heterogeneous among subjects included in GWASs. Therefore, annotating SNPs among the mid-hanging fruit for functionality provides more confidence to these findings, a more complete picture of the genetic architecture of asthma, and a model for prioritizing these loci for further studies.

Although our study provides novel observations about epigenetic mechanisms of asthma risk alleles, there are some limitations. First, the sample sizes for the eQTL and meQTL studies were smaller than the sample sizes recommended by moloc (*n*_min_=300) [47]. In such cases, moloc can miss true colocalizations in QTL datasets. For example, an eQTL-GWAS pair may actually be an eQTL-meQTL-GWAS triplet that we were underpowered to detect. As a result, the eQTL-GWAS and meQTL-GWAS pairs that we identified could be eQTL-meQTL-GWAS triplets or we may have missed other colocalizations entirely. This may be evidenced by the fact that only a single meQTL colocalized with a GWAS SNP at the *TSLP* locus in the TAGC GWAS, while the same SNP, rs1837253, colocalized with three meQTLs, including two additional CpGs, in the childhood and adult onset asthma (Additional file 1: Fig. S7), representing potential contributors to asthma disease mechanisms that were missed in the TAGC GWAS. Additionally, the designation of the colocalized triplet with *ORMDL3* at the 17q12-21 locus as RV-specific is also likely related to the reduced power to detect the same colocalization in the vehicle-treated cells. The fact that the same SNP is an eQTL for *ORMDL3* in both vehicle- and RV-treated cells (Additional file 10: Table S4) and that the MR results indicated that methylation at cg18711369 mediated *ORMDL3* expression in both the vehicle- and RV-treatments (Table 4) suggests that this colocalization may not in fact be RV-specific. Nonetheless, the 46 unique colocalizations detected in our study are likely to be real as we were able to replicate many of those findings in two or all three GWASs, including those for *ERBB2* and *TSLP*, respectively (Additional file 10: Table S4). Future studies in larger samples will increase confidence in our findings. Second, we focused on one cell type (upper airway epithelium), two exposures (vehicle and RV), and one epigenetic mark (DNA methylation). It is possible that other asthma-relevant colocalizations are specific to other tissues or cell types or to other exposures or culture conditions, and that other epigenetic marks, such as those associated with chromatin accessibility, would be additionally informative. Finally, because of the limited sample size, we did not test for QTLs that differed between subjects with and without asthma or perform ethnic-specific analyses. As a result, we likely identified QTLs that are robust to disease status, and potentially to ethnicity. However, utilizing the largest sample possible to identify treatment-specific molecular QTLs increased our power to differentiate molecular responses to RV infection.

## Conclusions

We identified *cis*-eQTLs and *cis*-meQTLs in an AEC model of host cell response to RV and integrated those data with three large asthma GWASs to assign potential molecular mechanisms for variants associated with asthma. By combining enrichment studies, colocalization analysis, Mendelian randomization, and pcHi-C, we provide robust statistical and experimental evidence of epigenetic mechanisms in upper airway cells contributing to childhood onset asthma. We demonstrate that a multi-omics approach using a disease-relevant cell type and disease-relevant exposure allows prioritization of disease-associated GWAS variants and provides insight into potential epigenetic mechanisms of asthma pathogenesis.

## Supplementary Information

**Additional file 1: **Additional Figures describing the sample cohort and results from our analyses. Figure S1: Overview of the e/meQTL and co-localization studies in AECs treated with RV. Figure S2: PCA and k-means clustering of genotypes. Figure S3: PCA of gene expression in vehicle and RV-treated AECs. Figure S4: PCA of DNA methylation in vehicle- and RV-treated cultured AECs. Figure S5: Molecular QTLs highlighting atopic samples. Figure S6: Summary results for molecular QTL mappings. Figure S7: meQTLs at rs1837253 located in the first untranslated exon of the *TSLP* gene.

**Additional file 2: **Gene expression covariate correlation matrix of correlation coefficients (gx correlation), *p-*values (gx pval), and correlation tests (gx test).

**Additional file 3: **Gene expression PCA results for unadjusted (Gx Unadjusted) and adjusted (Gx Adjusted) data. *P-*values are shown for correlations for the first 10 PCs with each covariate.

**Additional file 4: **DNA methylation covariate correlation matrix of correlation coefficients (DNA methylation correlation), *p-*values (DNA methylation pval), and correlation tests (DNA methylation test).

**Additional file 5: **DNA methylation PCA results for unadjusted (DNA methylation Unadjusted) and adjusted (DNA methylation Adjusted) data. *P-*values are shown for correlations for the first 10 PCs with each covariate.

**Additional file 6:.** FastQTL eQTL mapping results for vehicle-treated AECs (FDR< 0.05).

**Additional file 7:.** FastQTL eQTL mapping results for RV-treated AECs (FDR< 0.05).

**Additional file 8:.** FastQTL meQTL mapping results for vehicle-treated AECs (FDR< 0.05).

**Additional file 9:.** FastQTL meQTL mapping results for RV-treated AECs (FDR< 0.05).

**Additional file 10: **Tables showing enrichment and colocalization results from this study. Table S1: Interaction model results for genotype x atopy and genotype x steroid use for eQTLs and meQTLs. Table S2: Enrichment estimates of eQTLs for TAGC asthma GWAS SNPs from six tissues. No *P-*values were significant after FDR correction. Table S3: Enrichment estimates of eQTLs for adult onset asthma GWAS SNPs from six tissues. No *P-*values were significant after FDR correction. Table S4: moloc results for molecular QTL-GWAS pairs and triplets. Table S5: Asthma GWAS risk allele effects on gene expression and DNA methylation.

**Additional file 11:.** Supplementary methods describing the replication analysis of the molecular QTLs. Table S6: Percent of overlapping eQTL results with the URECA study. Table S7: Percent of overlapping meQTL results with the URECA study.

**Additional file 12:.** eQTL mash results (lfsr< 0.05).

**Additional file 13:.** meQTL mash results (lfsr< 0.05).

## Data Availability

RNAseq and DNA methylation datasets supporting the conclusions of this article are available in the Gene Expression Omnibus (GEO) repository under the accession number GSE172368 (https://www.ncbi.nlm.nih.gov/geo/query/acc.cgi?acc=GSE172368) [104]. The imputed genotypes have been deposited in the European Variation Archive (EVA) under the accession number PRJEB47290 (https://wwwdev.ebi.ac.uk/eva/?eva-study=PRJEB47290) [105].
