# Supplementary material for: Multi-omics colocalization with genome-wide association studies reveals a context-specific genetic mechanism at a childhood onset asthma risk locus
Source: Genome Med. 2021 Oct 10;13:157. doi: 10.1186/s13073-021-00967-y (PMC8504130; doi:10.1186/s13073-021-00967-y)
Supplement: Supplementary file 1 — Additional file 1: Additional Figures describing the sample cohort and results from our analyses. Figure S1: Overview of the e/meQTL and co-localization studies in AECs treated with RV. Figure S2: PCA and k-means clustering of genotypes. Figure S3: PCA of gene expression in vehicle and RV-treated AECs. Figure S4: PCA of DNA methylation in vehicle- and RV-treated cultured AECs. Figure S5: Molecular QTLs highlighting atopic samples. Figure S6: Summary results for molecular QTL mappings. Figure S7: meQTLs at rs1837253 located in the first untranslated exon of the TSLP gene. [file 13073_2021_967_MOESM1_ESM.pdf]

A

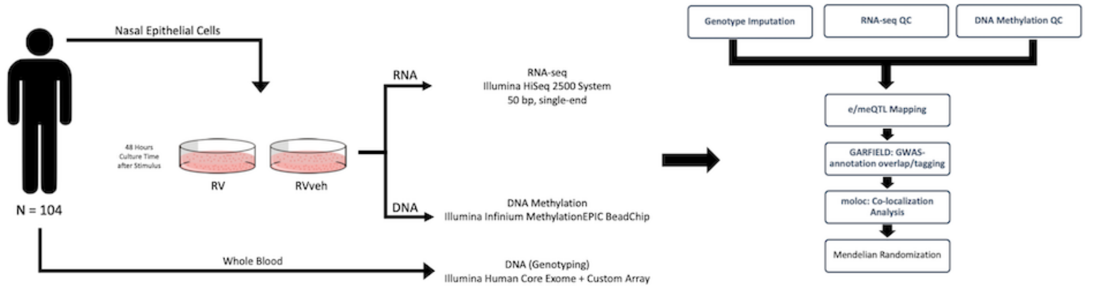

B

Sample composition

QTL Study	Combined	Gene Expression	DNA Methylation
Sample Size (N)	104	95	103
Gender (% Female)	39	41	33
Mean Age (Range)	44 (18-73)	45 (18-73)	45 (18-73)
% Asthma (Ever)	41	44	41
%CRSwNP	47	50	47
%Atopic	51	53	50

C

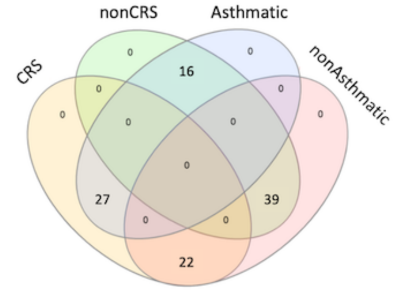

Fig. S1 | Overview of the e/meQTL and co-localization studies in AECs treated with RV. **(A)** Experimental design used to identify treatment-specific e/meQTLs in upper AECs from 104 individuals. **(B)** Sample composition. **(C)** Venn diagram showing the overlap of asthma and CRS status of the 104 study participants.

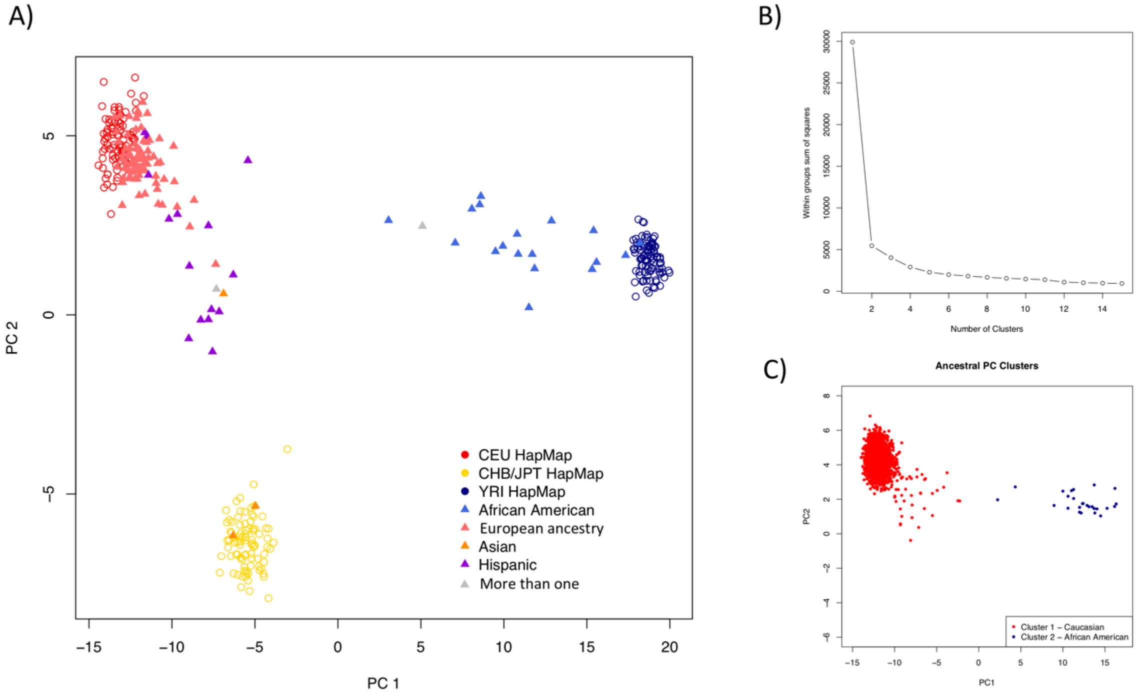

Fig. S2 | PCA and k-means clustering of genotypes. (A) PCA plot of study participant's genotypes (circles) projected on HapMap genotypes (squares). **(B)** Scree plot of k-means clustering of ancestral PCs in which the within groups sum of squares (y-axis) is plotted against the number of potential group clusters (x-axis); using the ‘elbow criterion’, it is determined that two clusters are best representative of how many clusters study samples can be grouped into for imputation. **(C)** PCA plot of study participants grouped into two cluster for genotype imputation, European (red), and African American (Blue), according to the k-means clustering criterion.

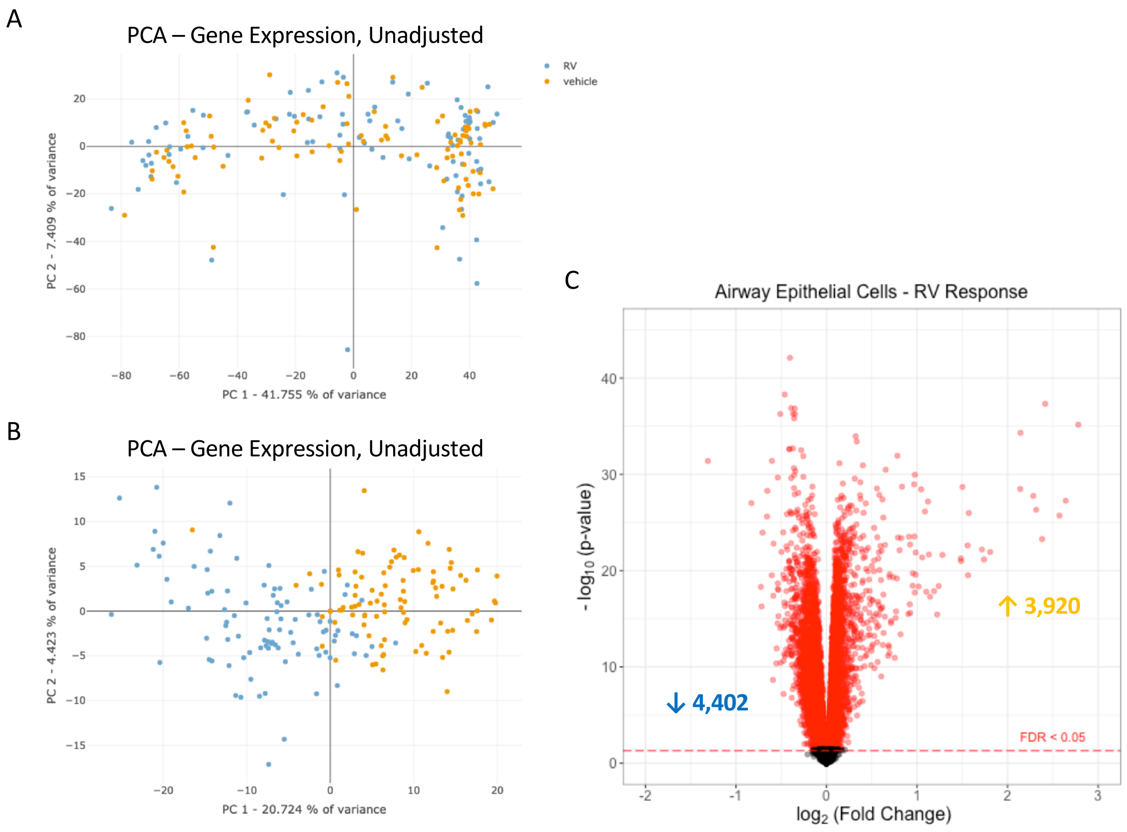

Fig. S3 | PCA of gene expression in vehicle and RV-treated AECs. (A) PCA plot of epithelial cell gene expression from 95 individuals treated with vehicle and RV before regressing out covariates. **(B)** PCA plot of gene expression in vehicle- and RV-treated cells after regressing out covariates. **(C)** Volcano plot showing treatment responses in epithelial cells were detected in the combined sample with 8,322 differentially expressed genes identified at a FDR≤0.05.

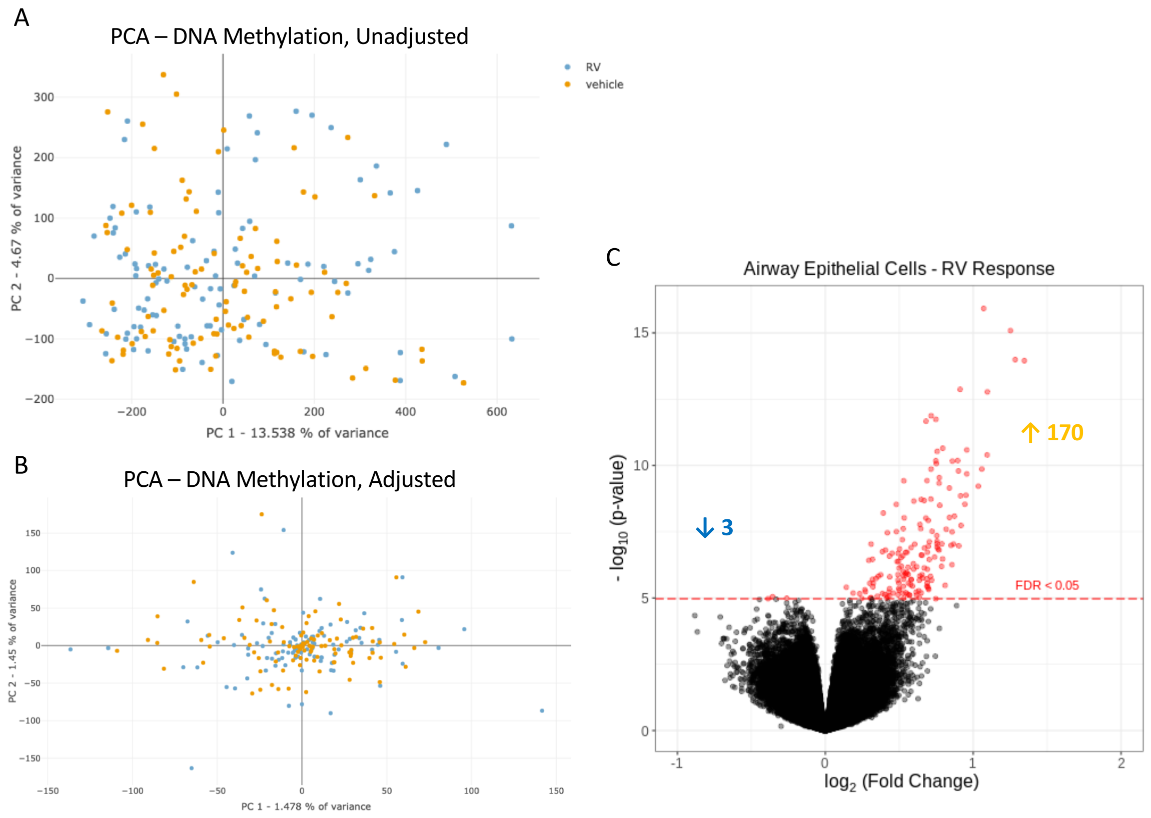

Fig. S4 | PCA of DNA methylation in vehicle- and RV-treated cultured AECs. (A) PCA plot of cultured airway epithelial DNA methylation from 103 individuals treated with vehicle and RV before regressing out covariates. **(B)** PCA plot of DNA methylation in vehicle- and RV-treated cells after regressing out covariates. Tables showing p-values of correlation with PCs and covariates before **(C)** Volcano plot showing treatment responses were detected in the combined sample with 173 differentially methylated CpGs at a FDR<0.05.

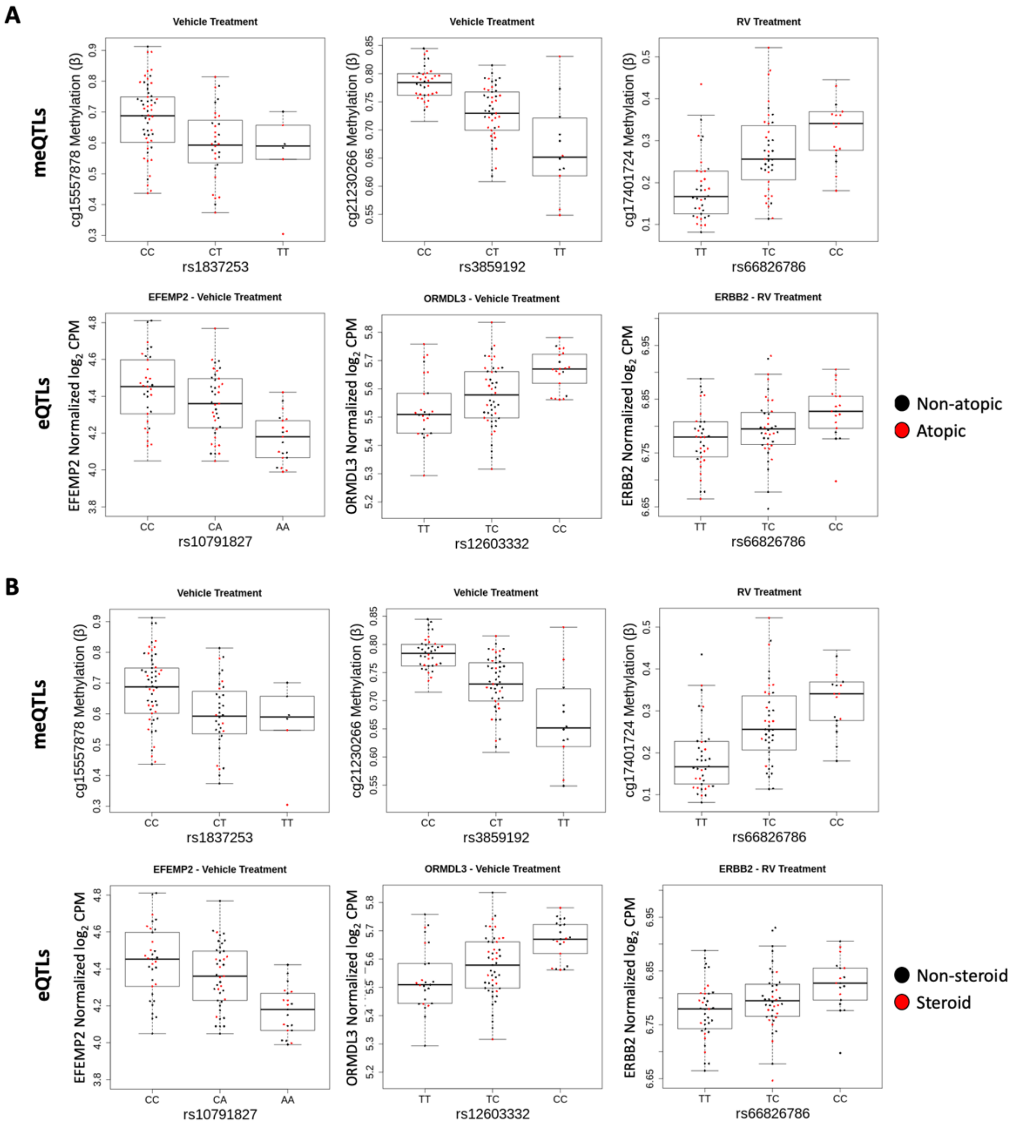

Fig. S5 | Molecular QTLs highlighting atopic samples. Box plots showing examples of eQTLs and meQTLs from vehicle- and RV-treated AECs highlighted in this study. A quality control measure showing that genotype-specific gene expression and DNAm levels were similar between the atopic and non-atopic individuals (A) and between individuals who used steroid medications vs those who did not (B). The box plots show that the molecular QTLs highlighted in this study were not confounded by either atopy or steroid use.

A

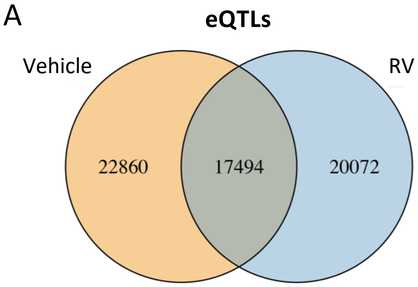

B

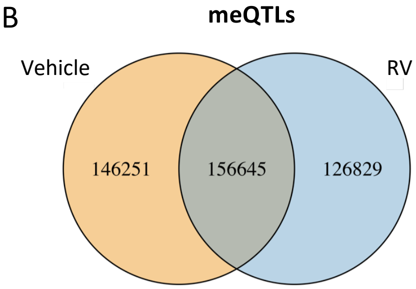

C

eQTL and meQTL mapping results (FDR<0.05)

	Vehicle	RV
**eQTLs**	40.354	37,566
SNPs	38,461	35,989
Genes	1,174	1,043
**meQTLs**	302,896	283,474
SNPs	219,905	206,583
CpGs	26,644	25,418

Fig. S6 | Summary results for molecular QTL mappings. Venn diagrams of eQTLs **(A)** and meQTLs **(B)** in each condition (FDR<0.05). **(C)** Summary of eQTL and meQTL mapping results for each treatment condition. The number of SNPs associated with the gene expression of at least one gene or CpG and the number of genes or CpGs whose expression or DNA methylation levels was associated with at least one SNP.

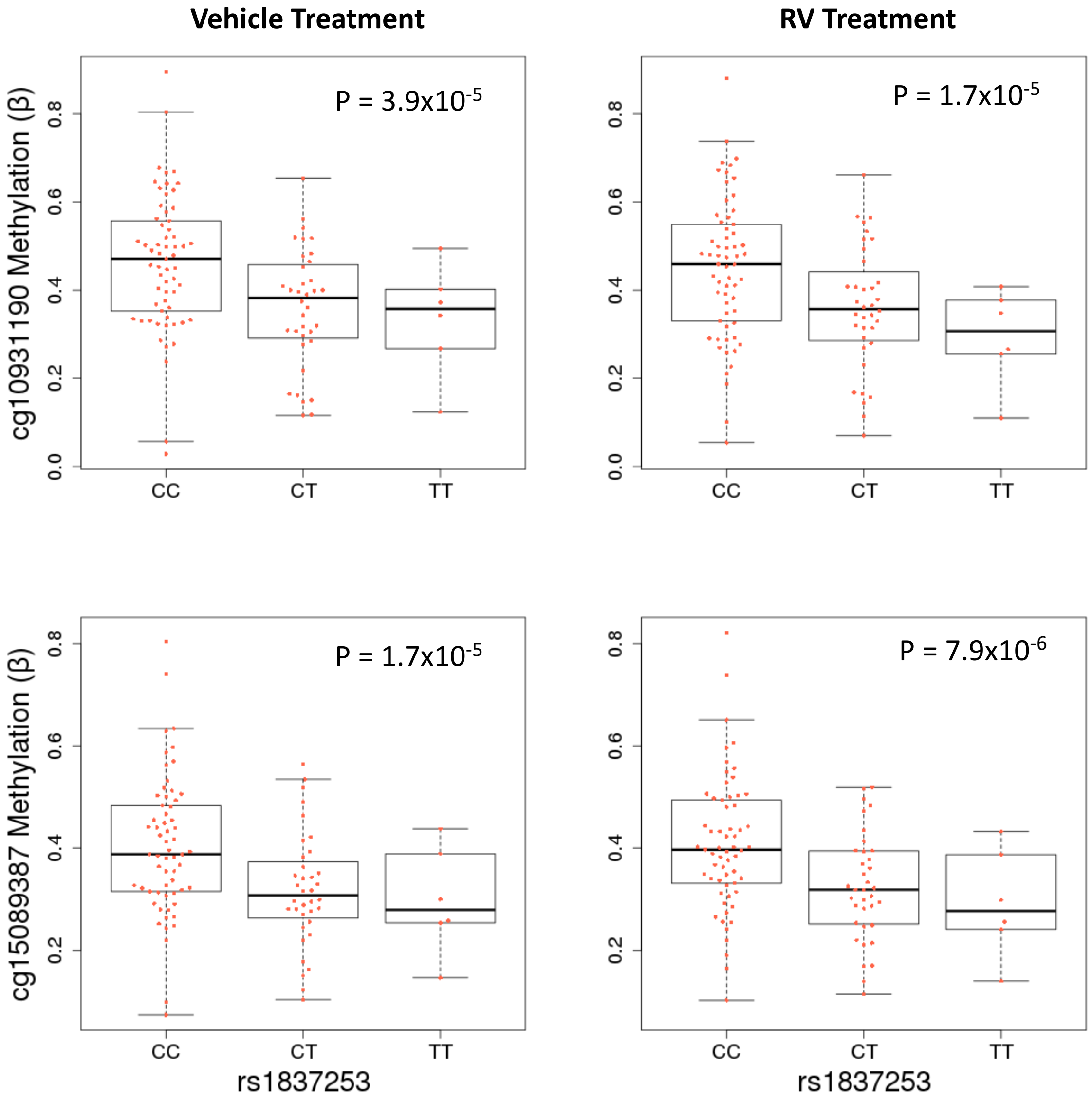

Fig. S7 | meQTLs at rs1837253 located in the first untranslated exon of the *TSLP* gene. Box plots of two meQTLs that were identified in both the vehicle- (left) and RV-treated (right) AECs were co-localized with the asthma risk variant (rs1837253) in adult onset and childhood onset asthma GWASs.
