# Supplementary material for: Multi-omics colocalization with genome-wide association studies reveals a context-specific genetic mechanism at a childhood onset asthma risk locus
Source: Genome Med. 2021 Oct 10;13:157. doi: 10.1186/s13073-021-00967-y (PMC8504130; doi:10.1186/s13073-021-00967-y)
Supplement: Supplementary file 11 — Additional file 11:. Supplementary methods describing the replication analysis of the molecular QTLs. Table S6: Percent of overlapping eQTL results with the URECA study. Table S7: Percent of overlapping meQTL results with the URECA study. [file 13073_2021_967_MOESM11_ESM.pdf]

Supplementary Methods

Replication analyses of molecular QTLs

Replication analyses of the molecular QTLs were performed with molecular QTL datasets in *ex vivo* airway epithelial cells that were available from other studies. First, we tested for overlap between our results in cultured AECs and *cis*-eQTLs and -meQTLs for genes at asthma GWAS loci in *ex vivo* nasal epithelial cells from 477 Puerto Rican children described by Kim et al. [1]. This study differed from ours in three important ways. First, Kim et al. used a different genotyping platform than the one used in our study (HumanOmni 2.5 and HumanCore Exome+Custom Array, respectively; both Illumina), and their eQTL studies focused on candidate regions whereas ours was genome-wide. This resulted in little overlap between SNPs that were eQTLs in our study and SNPs that were included in their study. Second, different DNAm arrays were used in their study and ours (Human Methylation 450k array and the EPIC array, respectively; both Illumina). Moreover, Kim et al. focused only on SNPs associated with expression for genes through DNA methylation at asthma loci, whereas our study was genome-wide, also resulting in very little overlap between SNPs that were meQTLs in the two studies. Third, because Kim et al. was performed in Puerto Rican children, we could not use LD surrogates for significant SNPs because of the different LD patterns in our populations.

The Kim et al. reported 1,150 eQTLs (for 55 genes at asthma loci) and 509 meQTLs that mediated the effects of the eQTLs. We did not have genotypes for any of the 1,150 reported eQTL SNPs. Therefore, we instead looked to see if any eQTLs from the 55 genes could be detected in our study. Of the 55 eGenes in the Kim et al. study, 28 were expressed in cultured cells, possibly reflecting composition differences between cultured and *ex vivo* cells, and 14 of those (50%) were eGenes in our study (lfsr≤0.05). Of the 240 CpG sites that mediated the effects

of the eQTLs at asthma loci in the Kim et al. study, 205 were on the EPIC array and passed QC in our study. Of those 205 meCpGs, 113 (55%) were meCpGs in our study. Therefore, we replicated ~50% of eGenes and meCpGs for which we had informative data.

We also compared our results to unpublished eQTLs (n=324; 75% African American [AA], 17% Hispanic, 7% of other ancestral origins, and 1% white) and meQTLs (n=246; 73% AA, 20% Hispanic, and 7% of other ancestral origins) in *ex vivo* nasal epithelial cells from 11-year old children in the Urban Environment and Childhood Asthma (URECA) birth cohort study [2]. For this study, we had available genotypes from whole genome sequences and performed DNA methylation studies using the EPIC array. As a result, all SNPs that were QTLs and all CpGs that were meCpGs in the current study were also available in the URECA data set. In these data, we used the same criteria for calling eQTLs and meQTLs as we did in the cell culture studies (i.e., ± 10 kb *cis* windows for meQTL mapping and ± 1 Mb *cis* windows for eQTL mapping). We used linear models to test for both eQTLs and meQTLs in URECA, including as covariates sex, recruitment site, ancestry PCs 1-3, epithelial cell proportions and latent factors (to adjust for unwanted variation; 7 and 2 latent factors for the eQTLs and meQTLs, respectively). We used an FDR of 0.05 in each study. We looked to see how many of the SNP-gene or SNP-CpG pairs that were QTLs in our study were also eQTLs or meQTLs in the URECA data, as well as whether the eGenes or meCpG and eSNP and meSNPs overlapped between the two studies.

The percentages of eQTLs (SNP-gene pairs; 1 Mb windows) in the cell culture study that were also eQTLs in the URECA data (FDR <0.05) are shown in Table S6. In these comparisons, approximately 19-20% of eQTLs and 41-43% of eGenes were replicated. The latter is similar to the comparisons with the Kim et al. study described above.

Table S6. Percent of overlapping eQTL results with the URECA study.

	Vehicle Treatment			RV Treatment		
	eQTL	eSNP	eGene	eQTLs	eSNP	eGene
% Overlap w/ URECA Study	19.1	23.3	41.8	20.5	24.7	43.6

The percentages of meQTLs (SNP-CpG pairs; ± 10 kb windows) in our cell culture study in each treatment that were also meQTLs in the URECA data (FDR<0.05) are shown in Additional File 10: Table S7. Just over 61% of meQTLs identified in each treatment group in our study were replicated in the URECA sample (FDR <0.05). Greater proportions of meSNPs and meCpGs in our study were replicated as meSNPs (72% and 75%) and meCpGs (77% and 81%) in the URECA data. The larger replication of meSNPs and meCpGs compared to meQTLs likely reflects the different LD patterns between the two studies due to the different racial/ethnic compositions of each study. We also observed significant correlations between beta values of replicated meQTLs in each dataset ($R^2 = 0.534$; $P=2.2x10^{-16}$), in which the direction of effect was the same in 82% of the data.

Table S7. Percent of overlapping meQTL results with the URECA study.

	Vehicle Treatment			RV Treatment		
	meQTL	meSNP	meCpG	meQTLs	meSNP	meCpG
% Overlap w/ URECA Study	61.2	76.9	77.3	61.4	72.5	81.0

Finally, we examined the overlap between the colocalized eQTLs and meQTLs. For these comparisons, we focused on meCpGs and eGenes because the colocalization with GWAS loci may select one of potentially many SNPs that are meQTLs or eQTLs for the same CpG site or gene. Of the 17 meQTL-GWAS pairs, 13 were meCpGs in the URECA dataset. Of the 5, eQTL-gene pairs, one was an eGene. Of the 24 triplets (eQTL-meQTL-GWAS), the meCpGs were replicated in 13 (eGenes in none).

References

1. Kim S, Forno E, Yan Q, Jiang Y, Zhang R, Boutaoui N, Acosta-Perez E, Canino G, Chen W, Celedon JC: **SNPs identified by GWAS affect asthma risk through DNA methylation and expression of cis-genes in airway epithelium**. *Eur Respir J* 2020, **55**(4).

2. Altman MC, Calatroni A, Ramratnam S, Jackson DJ, Presnell S, Rosasco MG, Gergen PJ, Bacharier LB, O'Connor GT, Sandel MT *et al*: **Endotype of allergic asthma with airway obstruction in urban children**. *J Allergy Clin Immunol* 2021.
